# Synthesis and Evaluation of [^18^F]FEtLos and [^18^F]AMBF_3_Los as Novel ^18^F-Labelled Losartan Derivatives for Molecular Imaging of Angiotensin II Type 1 Receptors

**DOI:** 10.3390/molecules25081872

**Published:** 2020-04-18

**Authors:** Martha Sahylí Ortega Pijeira, Paulo Sérgio Gonçalves Nunes, Sofia Nascimento dos Santos, Zhengxing Zhang, Arian Pérez Nario, Efrain Araujo Perini, Walter Miguel Turato, Zalua Rodríguez Riera, Roger Chammas, Philip H. Elsinga, Kuo-Shyan Lin, Ivone Carvalho, Emerson Soares Bernardes

**Affiliations:** 1 Radiopharmacy Center, Nuclear and Energy Research Institute (IPEN/CNEN-SP), CEP 05508-000 São Paulo, Brazil; msopijeira2015@usp.br (M.S.O.P.); snsantos@usp.br (S.N.d.S.); apereznario@usp.br (A.P.N.); eaperini@ipen.br (E.A.P.); 2 School of Pharmaceutical Sciences of Ribeirão Preto, University of São Paulo (FCFRP–USP), CEP 14040-903 Ribeirão Preto, Brazil; paulosgn@fcfrp.usp.br (P.S.G.N.); carronal@usp.br (I.C.); 3 Department of Molecular Oncology, BC Cancer Research Centre, Vancouver, BC V5Z 1L3, Canada; zzhang@bccrc.ca (Z.Z.); klin@bccrc.ca (K.-S.L.); 4 School of Pharmaceutical Sciences–University of São Paulo-USP, CEP 05508-000 São Paulo, Brazil; turato@usp.br; 5 Departamento de Radioquímica, Instituto Superior de Tecnologías y Ciencias Aplicadas (InSTEC), Universidad de La Habana, CP 10400 La Habana, Cuba; zalua@instec.cu; 6 Department of Radiology and Oncology, Faculty of Medicine, University of São Paulo, CEP 01246903 São Paulo, Brazil; rchammas@usp.br; 7 Department of Nuclear Medicine and Molecular Imaging, University Medical Center Groningen, University of Groningen, EB79, PO box 30.001, 9700 RB Groningen, The Netherlands; p.h.elsinga@umcg.nl

**Keywords:** fluoroethyl-losartan, [^18^F]FEtLos, ammoniomethyltrifluoroborate-losartan, [^18^F]AMBF_3_Los, angiotensin II type 1 receptors, [^18^F]Fluoroethylation, ^18^F-^19^F isotopic exchange approach, in vitro assays, µPET imaging, renal autoradiography

## Abstract

Losartan is widely used in clinics to treat cardiovascular related diseases by selectively blocking the angiotensin II type 1 receptors (AT_1_Rs), which regulate the renin-angiotensin system (RAS). Therefore, monitoring the physiological and pathological biodistribution of AT_1_R using positron emission tomography (PET) might be a valuable tool to assess the functionality of RAS. Herein, we describe the synthesis and characterization of two novel losartan derivatives PET tracers, [^18^F]fluoroethyl-losartan ([^18^F]FEtLos) and [^18^F]ammoniomethyltrifluoroborate-losartan ([^18^F]AMBF_3_Los). [^18^F]FEtLos was radiolabeled by ^18^F-fluoroalkylation of losartan potassium using the prosthetic group 2-[^18^F]fluoroethyl tosylate; whereas [^18^F]AMBF_3_Los was prepared following an one-step ^18^F-^19^F isotopic exchange reaction, in an overall yield of 2.7 ± 0.9% and 11 ± 4%, respectively, with high radiochemical purity (>95%). Binding competition assays in AT_1_R-expressing membranes showed that AMBF_3_Los presented an almost equivalent binding affinity (K_i_ 7.9 nM) as the cold reference Losartan (K_i_ 1.5 nM), unlike FEtLos (K_i_ 2000 nM). In vitro and in vivo assays showed that [^18^F]AMBF_3_Los displayed a good binding affinity for AT_1_R-overexpressing CHO cells and was able to specifically bind to renal AT_1_R. Hence, our data demonstrate [^18^F]AMBF_3_Los as a new tool for PET imaging of AT_1_R with possible applications for the diagnosis of cardiovascular, inflammatory and cancer diseases.

## 1. Introduction

Angiotensin II (ANG II) is the effector peptide that exerts most of the physiological functions of the renin-angiotensin system (RAS), and binds with high affinity to two G-protein coupled receptors: angiotensin II type 1 (AT_1_) and angiotensin II type 2 (AT_2_) receptors. The RAS is well-known as a key regulator of blood pressure and fluid homeostasis [1]. Nevertheless, the deregulation of RAS is responsible for several pathologies such as cardiovascular [2,3,4], renal [5,6,7], brain [8,9,10,11] and cancer [12,13,14] disorders via ANG II/AT_1_R signaling.

**Figure 1:**
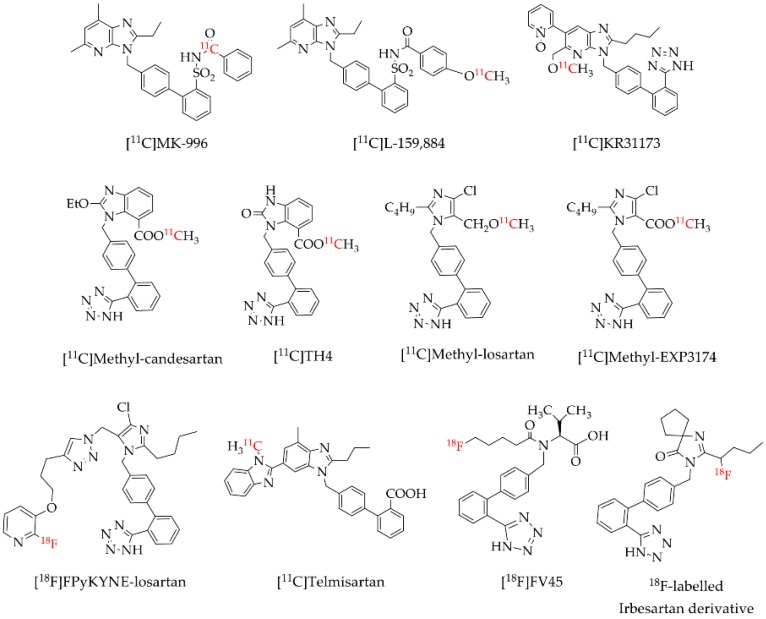
Reported AT_1_R positron emission tomography (PET) radioligands [15,16,17,18,19,20,21,22,23,24].

Positron emission tomography (PET) is a highly sensitive and non-invasive imaging modality widely used in nuclear medicine for the diagnosis and clinical monitoring of several diseases. PET imaging uses radiopharmaceuticals labelled with positron-emitting radioisotopes such as carbon-11 (^11^C) and fluorine-18 (^18^F). Several AT_1_R radioligands have been designed, synthesized and evaluated for molecular imaging of AT_1_ receptors [15,16,17,18,19,20,21,22,23,24]. Most of these AT_1_R radioligands (Figure 1), mostly derivatives from commercial angiotensin receptor blockers (ARBs), were labeled with ^11^C which is a drawback in the clinic practice due to the very short half-life of 20.4 min [25]. The use of the ^11^C-labelled radiopharmaceuticals demands an on-site cyclotron at the clinical PET center for their production in contrast to ^18^F-labelled radiopharmaceuticals. Compared to ^11^C, ^18^F has a lower maximum energy of the emitted positron of 635 keV, which favors the spatial resolution of PET images [25,26,27], and a higher half-life of 110 min [25] allowing the performance of PET studies without the need of an on-site cyclotron. Losartan is the most used selective AT_1_R blocker in the clinic among the ARBs, being the first choice for stroke, heart failure, diabetic nephropathy, hyperuricemia and erectile dysfunction, and also exerts potentially beneficial effects for cardiovascular prevention, atrial fibrillation, diabetes mellitus and cognitive decline [28]. However, to the best of our knowledge, just one ^18^F-labelled losartan-based AT_1_R radioligand ([^18^F]FPyKYNE-losartan; Figure 1) was developed so far. [^18^F]FPyKYNE-losartan was labeled with ^18^F using a time-consuming synthesis and presented a hydrophobic motif that induced a non-specific liver uptake when injected in vivo [21]. This prompted us to develop a novel ^18^F-labelled losartan derivative, more hydrophilic and easy to synthesize which could decrease in vivo non-specific uptake.

**Figure 2:**
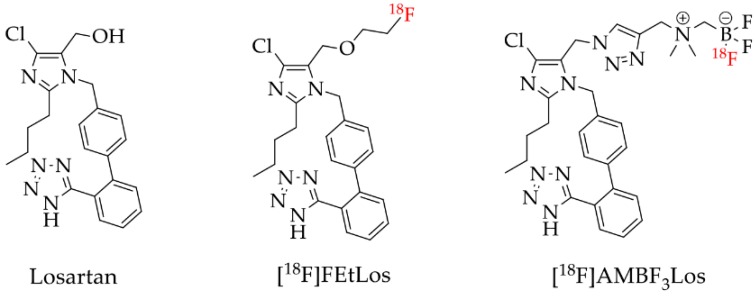
Losartan and the new ^18^F-labelled derivatives proposed in this work, [^18^F]FEtLos and [^18^F]AMBF_3_Los.

Therefore, here we described the synthesis and ^18^F-labeling of two novel losartan derivatives using less hydrophobic motifs such as: 2-fluoroethoxy and ammoniomethyltrifluoroborate (AMBF_3_), [^18^F]FEtLos and [^18^F]AMBF_3_Los, respectively (Figure 2).

The 2-[^18^F]fluoroethyl tosylate is a ^18^F-fluoroalkylating agent usually used to prepare PET tracers since it is easy to prepare with low volatility, high chemical stability and reactivity, and chemoselectivity [29,30]. Besides, the [^18^F]fluoroethyl group is considered as a surrogate for a [^11^C]methyl group because it can be coupled to the same functional groups [30]. Therefore, we designed the novel analog [^18^F]fluoroethyl-losartan ([^18^F]FEtLos; Figure 2) following a similar reaction scheme to the one reported for [^11^C]methyl-losartan (Figure 1).

On the other hand, the copper (I)-catalyzed Huisgen alkyne-azide 1,3-dipolar cycloaddition (CuAAC) is the most commonly used reaction for click chemistry, which has become a very attractive strategy in the design of PET tracers. This type of reaction shows an outstanding efficiency, regiospecificity and fast formation of the 1,4-disubstituted 1,2,3-triazoles [31] which are metabolically stable under physiological conditions [32,33]. In particular, the *N*-propargyl-*N*,*N*-dimethyl-ammoniomethyl-trifluoroborate is a hydrophilic linker that afford AMBF_3_-conjugated biomolecules using a CuAAC reaction. The AMBF_3_ motif allows an easy one-step ^18^F-labeling by ^18^F-^19^F isotope exchange reaction in acidic aqueous media using only nanomole amounts of the precursor. AMBF_3_ labeled products are usually very stable in vivo, present high molar activity and radiochemical purity, and do not depend on High Pressure Liquid Chromatography (HPLC) for purification [34,35,36]. Therefore, [^18^F]ammoniomethyltrifluoroborate-losartan ([^18^F]AMBF_3_Los; Figure 2) was also synthesized and characterized in the present work following the AMBF_3_ approach.

In addition, we studied the AT_1_R binding affinity of the newly synthetized derivatives both in vitro, ex-vivo and in vivo to evaluate its usefulness and pharmacokinetics as PET tracers for the noninvasive imaging of AT_1_R. We found that [^18^F]AMBF_3_Los presented the most suitable characteristics as an AT_1_R PET tracers: (1) its kit-like ^18^F-labeling approach was very fast and efficient, and (2) ^18^F-^19^F isotope exchange allowed the use of smaller amounts of the precursor than conventional radiolabeling methods.

## 2. Results

### 2.1. Chemistry

**Scheme 1:**
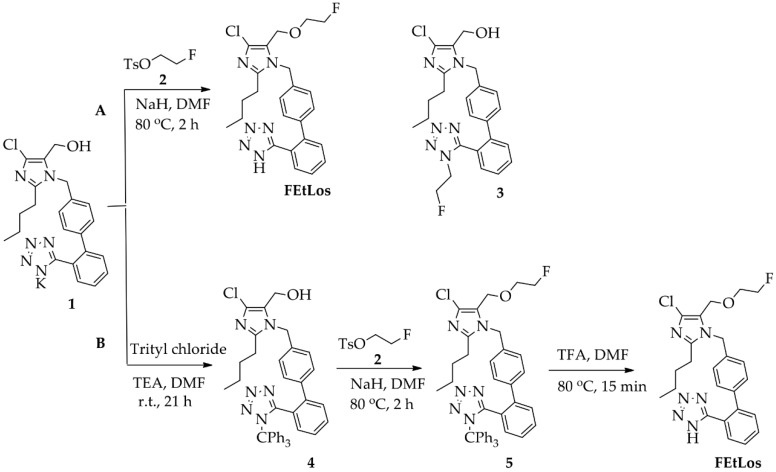
Synthesis of FEtLos ([^19^F]fluoroethyl-losartan, cold derivative) by *O*-alkylation of commercially available losartan potassium (**1**) using two strategies, (**A**) direct nucleophilic substitution of **1** with 2-fluoroethyl tosylate (**2**) or (**B**) a three steps procedure through protection of the *N*-tetrazol moiety of **1**, followed by *O*-alkylation and deprotection.

**Scheme 2:**
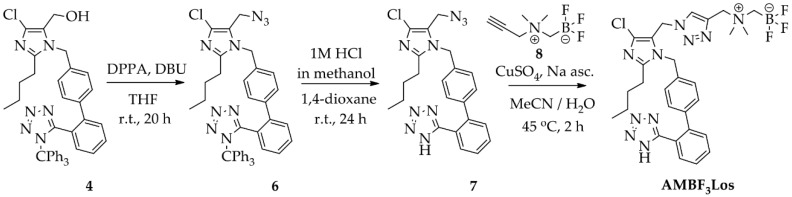
Synthesis of AMBF_3_Los ([^19^F]ammoniomethyltrifluoroborate-losartan) by click chemistry using a copper (I)-catalyzed azide-alkyne cycloaddition reaction.

The compounds 2-butyl-4-chloro-5-(2-fluoroethoxy)methyl-1-[(2′-(1*H*-tetrazol-5-yl)biphenyl-4-yl)methyl]-1*H*-imidazole (FEtLos) and 2-butyl-4-chloro-5-[((1*H*-1,2,3-triazol-4-yl)-(*N*,*N*-dimethyl-ammoniomethyl-trifluoroborate)methyl)methyl]-1-[(2′-(1*H*-tetrazol-5-yl)biphenyl-4-yl)methyl]-1*H*-imidazole (AMBF_3_Los) were synthesized as new derivatives of 2-butyl-4-chloro-5-hydroxymethyl-1-[(2′-(1*H*-tetrazol-5-yl)biphenyl-4-yl)methyl]-1*H*-imidazole (**1**) (losartan) with substitution at the imidazole C-5 position (Scheme 1 and Scheme 2).

The synthesis of the cold derivative FEtLos was carried out through an *O*-alkylation of the hydroxymethylene group at the C-5 position of losartan (**1**) with 2-fluoroethyl-tosylate (**2**) (Scheme 1A), readily prepared from the tosylation of 2-fluoroethanol using tosyl chloride diluted in pyridine [37]. Following the bimolecular nucleophilic substitution reaction (S_N_2), FEtLos was obtained with a yield of 26% while the sub-product **3** was obtained with a yield of 7% (FEtLos/**3** ratio = 3.7). Structural characterization of FEtLos was achieved by ^13^C NMR, ^1^H NMR, ^19^F NMR, (^1^H, ^13^C)-HMBC and HRMS analyses. In particular, the structure of FEtLos was confirmed based on the heteronuclear correlation over three bonds between the fluoroethyl methylene hydrogens (4.70 ppm) and the oxymethylene carbon (52.7 ppm) at the imidazole C-5 position (Figure S2). The compound **3** was also structurally characterized (Figure S3).

In order to improve the yield of FEtLos, we pursued an alternative synthetic route in which the *N*-tetrazole moiety of losartan was protected with a trityl group [20] to give 2-butyl-4-chloro-5-(hydroxymethyl)-1-[(2′-(1*H*-(1-(triphenylmethyl))tetrazol-5-yl)biphenyl-4-yl) methyl]-1*H*-imidazole intermediate (**4**) with a good yield (83%) (Scheme 1B). Then, the same *O*-alkylation reaction conditions of losartan (**1**) (NaH, 80 °C, 2 h) was applied for functionalization of **4**, originating the 2-butyl-4-chloro-5-(2-fluoroethoxy)methyl-1-[(2′-(1*H*-tetrazol-5-yl)biphenyl-4-yl) methyl]-1*H*-imidazole (**5**) as a single product with an overall yield of 33%. The *O*-alkylation reaction was also conducted at room temperature for 24 h, but compound **4** was not totally consumed, and the yield of compound **5** did not improve even with an excess of 2-fluoroethyl tosylate (**2**). Finally, acidic removal of the trityl protecting group of compound **5** in 1:40 trifluoroacetic acid (TFA)/*N*,*N*-dimethylformamide (DMF) solution at 80 °C for 15 min led to the formation of FEtLos with a low yield (15%) and several by-products due to the decomposition of **5**, mostly formed under prolonged reaction conditions. Therefore, the direct fluoroethylation of losartan (26% yield, Scheme 1A) without *N*-tetrazole protection was found to be a superior strategy to obtain FEtLos than the three-step synthetic route (4% overall yield, Scheme 1B).

For the preparation of AMBF_3_Los, a CuAAC reaction was chosen to introduce the *N*,*N*-dimethyl-ammonium methyl-trifluoroborate at the C-5 position of losartan through a triazole bridge to produce both the standard reference and the precursor of [^18^F]AMBF_3_Los by an ^18^F-^19^F isotopic exchange approach. Firstly, the trityl-protected compound **4** was treated with diphenyl phosphoryl azide (DPPA) to displace the primary hydroxyl group by azide and give 2-butyl-4-chloro-5-(azidomethyl)-1-[(2′-(1*H*-(1-(triphenylmethyl))tetrazol-5-yl)biphenyl-4-yl) methyl]-1*H*-imidazole (**6**) [21], with good a yield (77%). Before proceeding to the click reaction, the trityl group was removed in order to avoid hydrolysis of the final AMBF_3_Los trifluoroborate group (Scheme 2). Therefore, the intermediate (**7**) was obtained by acidic removal of trityl protecting group at room temperature for 24 h with an excellent yield (96%) using 1 M HCl in methanol and 1,4-dioxane. Finally, the coupling between losartan-derived azide **7** with the *N*-propargyl-*N*,*N*-dimethyl-ammoniomethyl-trifluoroborate (**8**), bearing the terminal acetylene group, by CuAAC reaction [34,36,38] under copper (I) catalyst at 45 °C for 2 h gave exclusively the 1,4-disubstituted regioisomer AMBF_3_Los in moderate yield (52%). Although the copper (I) catalyst was formed in situ by reduction of copper sulfate (II) in the presence of sodium ascorbate, ammonium hydroxide solution (NH_4_OH, 5%) was immediately added to this mixture to avoid copper (I) complex precipitation due to its poor water solubility [36]. The structural characterization of AMBF_3_Los was achieved by ^13^C-NMR, ^1^H-NMR, ^19^F-NMR, (^1^H, ^13^C)-HMQC, (^1^H, ^13^C)-HMBC and HRMS analyses. In particular, direct heteronuclear correlations H–C for the ammoniomethyltrifluoroborate group were showed: methylene hydrogens (4.30 ppm)-methylene carbon (58.9 ppm) linked to triazole and, methyl hydrogens (2.83 ppm)-methyl carbon (52.0 ppm) from de dimethylamine group, as well the direct heteronuclear correlations H–C for the triazole moiety (7.98 ppm, 126.7 ppm) and methylene group at imidazole C-5 position (5.25 ppm, 46.3 ppm) (Figure S5). Furthermore, the structure of AMBF_3_Los was confirmed based on the heteronuclear correlation over three bonds between the methylene hydrogens (5.24 ppm) at imidazole C-5 position and the tertiary carbon (125.3 ppm) from the triazole moiety (Figure S5).

### 2.2. Radiochemistry

**Scheme 3:**
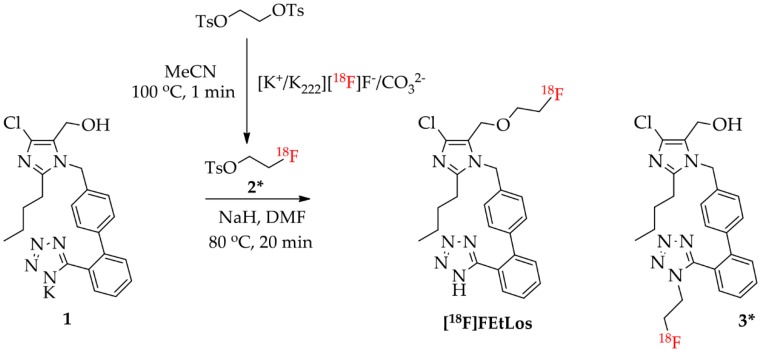
Radiosynthesis of [^18^F]FEtLos ([^18^F]fluoroethyl-losartan) by direct nucleophilic substitution of commercially available losartan potassium (**1**) with 2-[^18^F]fluoroethyl tosylate (2*), and formation of the isomer *N*-[^18^F]fluoroethyl-losartan (3*) as side-product.

**Scheme 4:**
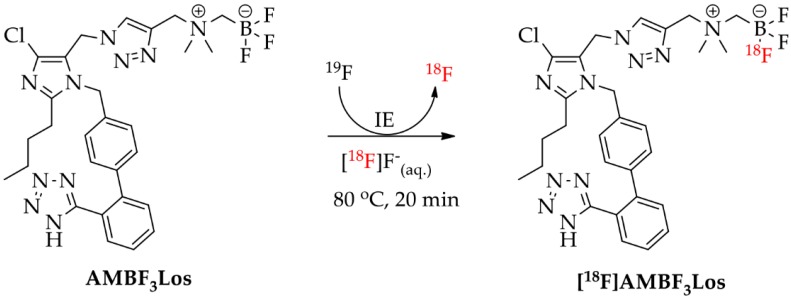
Radiosynthesis of [^18^F]AMBF_3_Los ([^18^F]ammoniomethyltrifluoroborate-losartan) by ^18^F-^19^F isotopic exchange (IE) reaction.

Scheme 3 and Scheme 4 show the synthetic route used for the preparation of the radiolabeled compounds [^18^F]FEtLos and [^18^F]AMBF_3_Los, respectively.

[^18^F]FEtLos was prepared by a ^18^F-fluoroalkylation of losartan using the prosthetic group 2-[^18^F]fluoroethyl tosylate (2*) (Scheme 3). Thus, 2* was manually prepared with a radiochemical yield of 30 ± 8% (decay-corrected from dried [^18^F]F^−^) and >99% chemical and radiochemical purity after HPLC-purification. The synthesis duration of 2* was of approximately 50 min. After ^18^F-fluoroalkylation of losartan potassium, [^18^F]FEtLos was purified by HPLC with a chemical and radiochemical purity >99% and its chemical identity was confirmed by HPLC co-injection of the cold compound (FEtLos) (Figure S6). [^18^F]FEtLos was prepared using a small amount of 2* and obtained with a radiochemical yield of 12 ± 5% (decay-corrected from 2*, approximately 100 min) and 1.4 ± 1.2 GBq (38 ± 33 mCi)/μmol molar activity (*n* = 10). The overall radiochemical yield was 2.7 ± 0.9% (decay-corrected from dried [^18^F]F^−^) with a total synthesis time of approximately three hours. The distribution coefficient of [^18^F]FEtLos at pH 7.4 (log D_7.4_) was 0.21 ± 0.09 (*n* = 3). The ^18^F-labeled side-product 3* was also formed by N-^18^F-fluoroalkylation of the losartan tetrazole moiety, confirmed by HPLC analyses of the crude reaction mixtures and compared with the HPLC profile of the cold compound **3**.

On the other hand, [^18^F]AMBF_3_Los was easily synthetized (35–55 min) by an ^18^F-^19^F isotopic exchange in an acidic aqueous medium, using only nanomole amounts of the AMBF_3_Los precursor and purified using a single Sep-Pak cartridge (Scheme 4). The manual radiosynthesis of [^18^F]AMBF_3_Los, using 25 nmol of precursor and low activities of [^18^F]F^−^, was achieved with a radiochemical yield of 11 ± 4%, a molar activity of 2.4 ± 0.5 GBq (0.06 ± 0.01 Ci)/µmol, and a radiochemical purity >97% (*n* = 20). When the radiosynthesis of [^18^F]AMBF_3_Los was done using 100-fold higher activities of [^18^F]F^−^ and 4-fold higher mass of the precursor (100 nmol), a radiochemical yield of 10 ± 1% with a radiochemical purity >97% and molar activity of 108 ± 29 GBq (2.9 ± 0.8 Ci)/µmol was achieved. The chemical identity of [^18^F]AMBF_3_Los was also confirmed by a single peak in the analytical HPLC profile after co-injection of the final formulation and the cold AMBF_3_Los, previously prepared (Figure S6). [^18^F]AMBF_3_Los displayed a log D_7.4_ of −0.43 ± 0.02 (*n* = 3).

### 2.3. In Vitro Binding Assays

**Figure 3:**
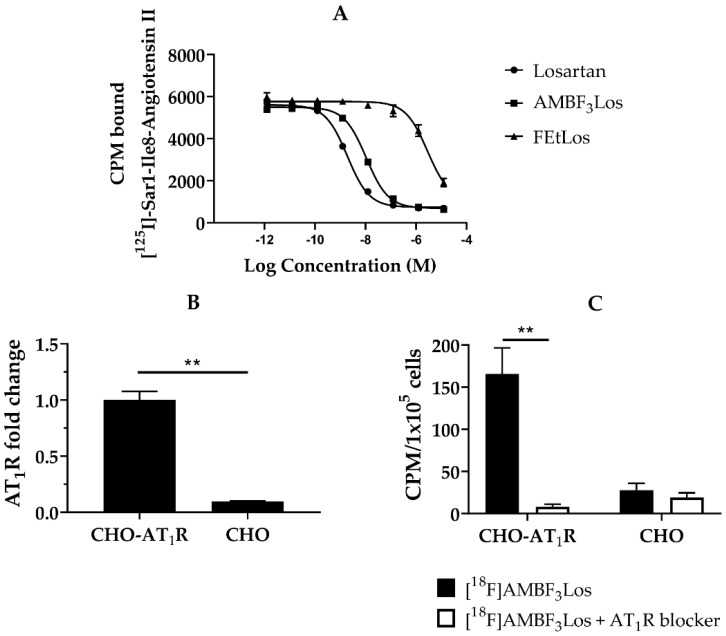
In vitro receptor binding assay. (**A**) A representative displacement curve of [^125^I]-(Sar1,Ile8)-Angiotensin II by losartan potassium, FEtLos and AMBF_3_Los respectively. (**B**) AT_1_ mRNA receptor abundance (*n* = 3) determined by RT-PCR in Chinese hamster ovary cells (CHO-AT_1_R, CHO); relative quantification was done using the ΔΔCt method normalizing to β-actin gene expression. (**C**) In vitro [^18^F]AMBF_3_Los uptake in CHO-AT_1_R or CHO cells seeded in 6-well plates after the tracer incubation for 60 min at 4 °C in the presence (white bars, *n* = 3) or absence (black bars, *n* = 3) of the AT_1_R blocker losartan potassium (100 µM/well). Graphs show the mean ± standard deviation (SD) of three independent experiments (*n* = 3). Data were analyzed by one unpaired *t*-test (multiple *t* tests); ** *p* < 0.01 (Holm-Sidak method).

The AT_1_R receptor affinities for Losartan, AMBF_3_Los and FEtLos were evaluated using the human AT_1_R membrane preparations and [^125^I]-(Sar1,Ile8)-Angiotensin II as the radioligand. The Ki values of Losartan potassium, AMBF_3_Los and FEtLos were 1.5 ± 0.3 nM (*n* = 3), 7.9 ± 0.4 nM (*n* = 3) and 2.2 ± 0.2 μM (*n* = 3) (Figure 3A). Since AMBF_3_Los showed the strongest AT_1_R receptor binding affinity, we further evaluated [^18^F]AMBF_3_Los.

We next evaluated the in vitro cellular uptake of [^18^F]AMBF_3_Los in CHO-AT_1_R cells, which overexpress the human AT_1_R (Figure 3B), and CHO cells (control). As observed in Figure 3C, CHO-AT_1_R cells presented a higher uptake of [^18^F]AMBF_3_Los in comparison to CHO cells. Incubation of 100 µM of losartan potassium blocked the CHO-AT_1_R uptake, indicating that the binding of [^18^F]AMBF_3_Los was receptor-specific (*p* < 0.01). [^18^F]FEtLos, on the other hand, did not show any specific AT_1_R binding for CHO-AT_1_R cells, which is in agreement with the low Ki value (Figure S7).

### 2.4. In Vivo Assays

**Figure 4:**
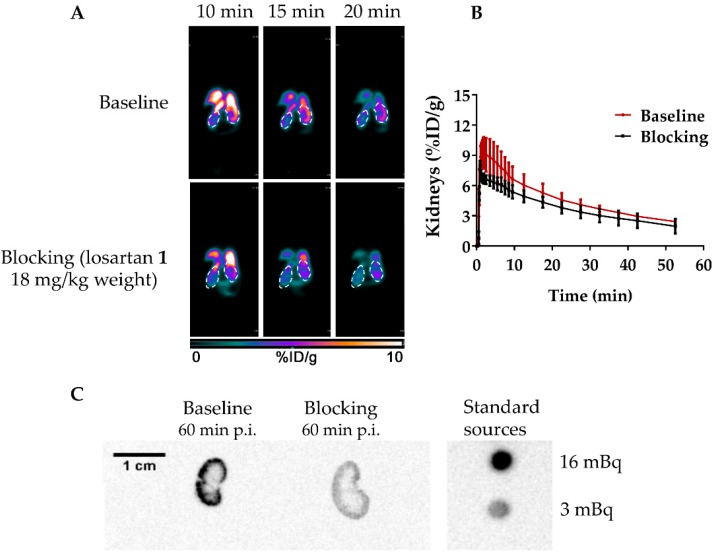
In vivo biodistribution of [^18^F]AMBF_3_Los. (**A**) Representative frames (10, 15 and 20 min) of the dynamic PET imaging (coronal view, 0–10% ID/g) in healthy NOD.Cg-Prkdc^scid^Il2rg^tm1Wjl^/SzJ mice of the baseline and AT_1_R blocked groups (co-injection with losartan, 18 mg/kg). (**B**) The time-activity curves obtained from the dynamic PET data at baseline (*n* = 3) and AT_1_R blocked (*n* = 3) groups. (**C**) Representative autoradiography images of kidneys from healthy mice at baseline and AT_1_R blocked groups after the 60 min dynamic PET scans.

A dynamic µPET study for healthy NOD.Cg-Prkdc^scid^Il2rg^tm1Wjl^/SzJ mice were next performed with [^18^F]AMBF_3_Los (Figure 4A,B). The µPET dynamic scan at baseline showed a high [^18^F]AMBF_3_Los uptake in the mouse renal cortex at early time points (10, 15 and 20 min) that was reduced when losartan potassium (AT_1_R blocker) was co-injected with [^18^F]AMBF_3_Los (Figure 4A). To confirm this finding, a volume of interest (VOI) analysis was performed on the reconstructed images to generate the time-activity curves (TAC) for [^18^F]AMBF_3_Los (Figure 4B), which showed renal tracer uptake within the first few minutes, and the activity was slowly washed out. In contrast, the presence of losartan potassium (AT_1_R blocker) reduced renal uptake during the first 10 min.

To validate the µPET imaging studies, a biodistribution study of [^18^F]AMBF_3_Los was performed at 60 min post injection (p.i.) as summarized in Table 1. No significant differences were observed compared to the µPET imaging quantification for the renal uptake. The high [^18^F]AMBF_3_Los activity uptake in intestines suggests it may also be cleared through the hepatobiliary system.

Autoradiographies of the mouse kidneys were also taken after the 60-min dynamic µPET scan with [^18^F]AMBF_3_Los showing that [^18^F]AMBF_3_Los was mainly captured in the kidney cortex, and this uptake was blocked by the co-injection of losartan potassium (Figure 4C). Indeed, the renal cortex activity was approximately 5-times smaller in the kidney of the mouse previously blocked with losartan potassium in comparison to the unblocked one. Thus, these data suggest that [^18^F]AMBF_3_Los specifically binds to renal AT_1_R.

The renal AT_1_R binding specificity of [^18^F]FEtLos was also evaluated by an ex vivo µPET/CT imaging of Balb/c Nude mice kidneys 10 min post injection (p.i.) of the tracer. As shown in Figure S8, [^18^F]FEtLos showed a high uptake in the kidneys (baseline), which was blocked when losartan potassium (AT_1_R blocker) was co-injected with [^18^F]FEtLos, despite the low affinity of the radiotracer. These positive results of the ex vivo renal AT_1_R binding of [^18^F]FEtLos might be possible due to the high AT_1_R density in the kidneys, that allows visualizing specific uptake in spite of the low AT_1_R binding affinity of [^18^F]FEtLos.

## 3. Discussion

The renin-angiotensin system (RAS) plays a fundamental role in the control of the cardiovascular and renal systems. Recent evidences suggest that AT_1_R has been implicated in a few brain disorders and has been associated with cancer progression and prognosis. Therefore, the in vivo imaging of AT_1_R could provide a diagnostic and prognostic information for the management of several diseases.

Although a few PET AT_1_R radioligands have been evaluated for cardiovascular [39,40], renal [22,23,24,41,42,43,44] and brain [18,45] AT_1_R imaging, most of them were labeled with ^11^C, which brings some disadvantages over ^18^F in the clinical practice, such as the presence of an in-site cyclotron. Recently, it was reported the synthesis of [^18^F]FPyKYNE-losartan (Figure 1), a losartan-derivative containing a hydrophobic motif that could favor a non-specific liver uptake [21]. Therefore, here we described the design, synthesis and characterization of two new AT_1_R radiotracers: [^19/18^F]FEtLos and [^19/18^F]AMBF_3_Los using less hydrophobic motifs.

Both compounds were designed via the substitution of the hydroxyl group by fluoro ligands at the imidazole 5-position of losartan. Several substituents with different length such as a small methyl group [20] and a bulky side chain [46] have been introduced into the losartan pharmacophore at the same position. For instance, a few losartan derivatives were synthesized by adding nitric oxide (NO)-donor side chains to losartan at the imidazole 5-position (Figure S9), displaying antagonist potency values similar to losartan [47]. Besides, the chelate-coupled losartan-Leucine-Diglycoloyl-Tetraethyleneglycol-Tetraamine (Figure S10) showed superior AT_1_R affinity compared to parental losartan; radiolabeling of this compound with ^99m^Tc displayed an acceptable biodistribution profile in mouse model of post-myocardial infarction heart failure [46]. The increase for AT_1_R affinity of this losartan analog may be probably due to the higher possibility to form hydrogen bonds, which may increase their affinity to the receptor. In addition, the [^18^F]FPyKYNE-losartan derivative also showed a high affinity to kidney AT_1_R in rats and pigs [44].

[^18^F]FEtLos and the cold FEtLos were synthetized by both [^18/19^F]fluoroethylation of losartan potassium and tetrazole-protected losartan through a S_N_2 reaction. Many PET tracers have been synthesized using a two-step radiosynthesis because the direct [^18^F]fluorination is not feasible most of the time. Direct fluorination usually requires high temperatures and non-physiological pH that can harsh the precursors [27]. Hence, ^18^F-labeling using prosthetic groups, such as the 2-[^18^F]fluoroethyl tosylate (2*) for ^18^F-fluoroalkylation reactions [30], is commonly used in spite of the longer time of synthesis. Moreover, although the insertion of fluoroethylene group in the NH-tetrazole group is interesting, we decided to maintain the negatively-charged tetrazole moiety intact because of its crucial role for AT_1_R binding through hydrogen bridge interactions with basic amino acid residues of the receptor [48,49,50], its prolonged half-life and enhanced metabolic stability [51]. In addition, the tetrazolic ion is more stable than the alkoxide ion due to the spatial delocalization of the negative charge of the former, favoring reactive alkoxide formation and alkylation to produce FEtLos with a ratio of 3.7 with the corresponding NH-tetrazole isomer (**3**).

On the other hand, the cold AMBF_3_Los was synthetized via a copper (I)—catalyzed Huisgen alkyne—azide 1,3-dipolar cycloaddition, the most used click reaction; while the ^18^F-labelled compound was obtained from an aqueous ^18^F-^19^F isotopic exchange reaction. The cold AMBF_3_Los was afforded in similar yield to other compounds using the same approach [38,52]. The one step ^18^F-^19^F isotopic exchange approach using organotrifluoroborates resembles a ‘kit like” ^18^F-labeling method and requires much lower amount of precursor (nanomoles) that the conventional radiolabeling methods (micromoles of precursor). This approach was previously used for radiolabeling peptides using high activities of [^18^F]F^−^ and very low amounts of precursor (75–100 nmol), within 30 min with a radiochemical purity >99%, a high molar activity (68–148 GBq/µmol), a radiochemical yield of 20–28%, with HPLC-free purification [34,38,53]. Small [^18^F]AMBF_3_-conjugated molecules were also synthesized with a radiochemical yield of 14.8 ± 0.4% with a molar activity of 24.5 ± 5.2 GBq/μmol and radiochemical purity >99% [54]. Taking into account that the reaction medium is acid, a significant amount of [^18^F]F^−^ is lost by volatilization as [^18^F]hydrogen fluoride during the labeling reaction under vacuum and heating. According to the literature, reaction pH is the crucial step of this radiolabeling approach, and pH 2–2.5 generally gives lower by-product (boronic acid) levels and better radiochemical yield [36]. The carrier [^19^F]fluoride was used during the radiolabeling because it increases the [^18^F]BF_3_ formation while still providing a molar activity of 37 GBq/µmol [55].

Results of the in vitro receptor binding assays showed that only the derivative AMBF_3_Los displayed a good binding affinity to human AT_1_R compared to FEtLos. Therefore, the substitution of hydroxyl group by the 2-fluoroethoxy motif decreased the AT_1_R binding affinity due to the lack of hydrogen bonding or other intermolecular forces. The 1,2,3-triazole is a polar moiety and a good hydrogen-bond acceptor that may keep the receptor binding affinity of AMBF_3_Los, as reported for the [^18^F]FPyKYNE-losartan derivative, which also contains a 1,2,3-triazole at the imidazole 5-position and displays high affinity for AT_1_R (dissociation constant, K_D_ 49.4 nM) to rat kidney AT_1_R [44]. The structure-activity relationship at the imidazole 5-position revealed that it generally prefers small hydrogen-bonding substituents such as alcohols or carboxylic acids but will also tolerate a wide range of groups [48]. Hence, our results suggested that hydrogen-bonding substituents at the imidazole 5-position play an important role to AT_1_R binding affinity besides the tetrazole moiety.

Next, both in vitro and in vivo assays showed that [^18^F]AMBF_3_Los specifically binds to the AT_1_ receptors. Therefore, [^18^F]AMBF_3_Los could be further evaluated as a specific PET radioligand to image the AT_1_ receptors in disease, for instance, in cancer; whereas, the low binding affinity of [^18^F]FEtLos limits its use as a AT_1_R PET tracer. In addition, the log D_7.4_ measurements of [^18^F]AMBF_3_Los (−0.4) showed that this new derivative is more hydrophilic than the parental losartan with a log D_7.4_ of 1.7 [56]. As such, we could observe that [^18^F]AMBF_3_Los displayed a smaller liver uptake (% ID/g = 0.5) than the previously reported derivative [^18^F]FPyKYNE-losartan (% ID/g = 66.7) in vivo at the same time-point (60 min) [44]. Therefore, [^18^F]AMBF_3_Los exhibited a significant less non-specific liver uptake compared to the reported [^18^F]FPyKYNE-losartan.

In particular, the PET imaging of AT_1_R in cancer has not been explored so far despite the upregulation of AT_1_R protein levels in human cells and tumor tissues of breast, prostate, gastric, bladder, ovarian and endometrial-derived cancers compared to non-cancerous tissues [57,58,59,60,61,62]. Accordingly, losartan has been shown to inhibit breast [63], gastric [62] and ovarian [64] cancer development and progression suggesting that AT_1_R could be a potential therapeutic target to complement current cancer treatment. Hence, [^18^F]AMBF_3_Los as AT_1_R radioligand for PET imaging can be a very useful tool for assessing AT_1_R-positive tumors.

## 4. Materials and Methods

### 4.1. General Methods

All the chemicals and solvents were purchased with analytical grade from commercial sources and used without further purification. All the cold compounds were characterized by high resolution mass spectrometry (HRMS, Billerica, MA, USA) and nuclear magnetic resonance (NMR) experiments, recorded on a Bruker Daltonics micrOTOF-Q II ESI-Qq-TOF spectrometer and Bruker Avance DRX 300, DPX 400, DPX 500 equipments (Billerica, MA, USA). Chemical shifts (δ) were reported in parts per million (ppm) relative to an internal tetramethylsilane (TMS) standard. Coupling constants (*J*) were reported in Hertz (Hz). No carrier-added [^18^F]F^−^ was produced by the ^18^O(p,n)^18^F reaction using enriched water (H_2_^18^O) target in a 18-MeV cyclotron (IBA, Belgium) using H_2_^18^O target. Sep-Pak Light QMA cartridges were pre-conditioned with 10 mL of potassium carbonate 0.5 M (K_2_CO_3_), followed by 20 mL of Milli-Q water and purged with 20 mL air. µ-QMA cartridges were reused, and always flushed with 3 mL brine and 6 mL Milli-Q water before [^18^F]F^−^ trapping. Sep-Pak C18 Plus cartridges were preconditioned with 5 mL ethanol and 10 mL of Milli-Q water. Sep-Pak C18 Light cartridges were preconditioned with 3 mL ethanol and 3 mL of Milli-Q water. Radioactivity of [^18^F]F^−^ was measured using a Capintec radioisotope dose calibrator (CRC-15R, Ramsey, NJ, USA). HPLC system from Agilent Technologies (Santa Clara, CA, USA) was used to purifications and quality control of the [^18^F]FEtLos and [^18^F]AMBF_3_Los.

### 4.2. Chemistry

#### 4.2.1. Synthesis of 2-Butyl-4-chloro-5-(2-fluoroethoxy)methyl-1-[(2′-(1*H*-(1-(triphenylmethyl)) tetrazol-5-yl) biphenyl-4-yl)methyl]-1*H*-imidazole (**5**)

The compounds 2-fluoroetyl tosylate (**2**) [37] and tetrazole-protected losartan **4** [20], were synthetized according to the literature. The compound **2** (28 μL, 37 mg, 0.17 mmol), was dropwise added to an ice-cooled solution of **4** (50 mg, 0.11 mmol) and NaH (3.6 mg, 0.15 mmol) in anhydrous DMF (400 μL) under stirring and argon atmosphere. The reaction was set at 0 °C for 30 min, and then, at 80 °C for 2 h (yellow in color), and monitored by TLC (silica gel, toluene/ethyl acetate, 90:10, *R*_f_ = 0.5). Afterwards, the reaction was quenched by dripping deionized water, and the crude product was extracted by ethyl acetate (3 times) using brine as aqueous phase. The organic layers were pool together, dried over magnesium sulphate (MgSO_4_), filtered, and the solvent was evaporated. Residue was purified by flash column chromatography (silica gel, toluene/ethyl acetate, 90:10, *R*_f_ = 0.5) to afford 17.8 mg (33%) of **5** as a yellow oil. ^1^H NMR (300 MHz, CDCl_3_) δ_H_ 7.93 (dd, *J* = 7.5 Hz, 1.5 Hz, 1H), 7.53–7.41 (m, 2H), 7.38–7.32 (m, 4H), 7.29–7.22 (m, 6H), 7.11 (d, *J* = 8.2 Hz, 2H), 6.95–6.90 (m, 6H), 6.75 (d, *J* = 8.3 Hz, 2H), 5.07 (s, 2H), 4.53–4.44 (m, 1H), 4.39–4.33 (m, 1H), 4.23 (s, 2H), 3.62–3.54 (m, 1H), 3.52–3.45 (m, 1H), 2.57–2.47 (m, 2H), 1.77–1.56 (m, 2H), 1.36–1.20 (m, 2H), 0.86 (t, *J* = 7.3 Hz, 3H). ^13^C NMR (75 MHz, CDCl_3_) δ_C_ 164.1, 149.0, 141.4, 141.0, 134.6, 130.9, 130.4, 130.3, 130.1, 130.0, 129.3, 129.2, 128.5, 128.1, 127.9, 127.8, 126.4, 125.4, 121.7, 83.6, 83.0, 82.0, 68.60, 68.4, 61.1, 47.3, 29.9, 26.9, 22.5, 13.9. ^19^F NMR (282 MHz, CDCl_3_) δ_F_ −223.16 (referred to the TFA reference standard). HRMS (ES^+^): *m*/*z* [M+H]^+^ calculated for C_43_H_41_ClFN_6_O: 711.3014; found: 711.3018.

#### 4.2.2. Synthesis of 2-Butyl-4-chloro-5-(2-fluoroethoxy)methyl-1-[(2′-(1*H*-tetrazol-5-yl)biphenyl-4-yl)methyl]-1*H*-imidazole (FEtLos)

Method A: A solution of losartan potassium **1** (100 mg, 0.22 mmol) in anhydrous DMF (500 μL) was added to an ice-cooled mixture of NaH (6 mg, 0.22 mmol) and anhydrous DMF (500 μL) under stirring and argon atmosphere. Then, a solution of 2-fluoroethyl tosylate (**2**) [37] (40.4 μL, 0.24 mmol) in anhydrous DMF (400 µL) was dropwise added into the solution previously prepared. The reaction mixture was stirred at room temperature for 30 min, and then, at 80 °C for two hours (orange in color). The progress of the reaction was monitored by TLC (silica gel, hexane/ethyl acetate, 50:50 and 30:70, *R*_f_ = 0.31 and 0.68 respectively). The reaction was quenched and the solvent was evaporated using a rotary evaporator with vertical dry-ice condenser. The residue was purified by flash column chromatography (silica gel, hexane/ethyl acetate, 50:50–30:70) to afford 26 mg of FEtLos (26%, light yellow oil) (Scheme 1A).

Method B: A solution of DMF-TFA 40:1 (64 μL) was added to the solution of **5** (8 mg, 11.3 µmol) in anhydrous DMF. After heating at 80 °C for 15 min, the reaction was monitored and the crude product was purified by semi-preparative HPLC (Shimadzu Corporation, Kyoto, Japan) with the conditions: Macherey-Nagel Nucleodur^®^ C18 column (250 × 10 mm, 10 μm), solvent A: 0.1% TFA water; solvent B: MeCN; 0–40 min, 10–100% A; 3 mL/min; 254 nm to afford 1.2 mg (15%) of compound FEtLos (Scheme 1B).

^1^H NMR (500 MHz, CDCl_3_) δ_H_ 7.88 (dd, *J* = 7.5 Hz and 0.8 Hz, 1H), 7.57–7.40 (m, 3H), 7.14 (d, *J* = 8.1 Hz, 2H), 6.94 (d, *J* = 8.0 Hz, 2H), 5.28 (s, 1H), 5.19 (s, 2H), 4.80 (s, 2H), 4.76–4.74 (m, 1H), 4.71–4.69 (m, 1H), 4.47 (s, 2H), 2.60–2.57 (m, 2H), 1.70–1.64 (m, 2H), 1.35 (h, *J* = 7.3 Hz, 2H), 0.88 (t, *J* = 7.3 Hz, 3H). ^1^H NMR (500 MHz, DMSO-*d*_6_) δ_H_ 7.77 (d, *J* = 7.1 Hz, 1H), 7.63 (t, *J* = 7.1 Hz, 1H), 7.55 (t, *J* = 7.1 Hz, 1H), 7.47 (d, *J* = 7.4 Hz, 1H), 7.09 (d, *J* = 8.0 Hz, 2H), 6.99 (d, *J* = 8.0 Hz, 2H), 5.75 (s, 1H), 5.25 (s, 2H), 4.97 (t, *J* = 4.8 and 23.1 Hz, 1H), 4.91 (t, *J* = 4.8 and 23.1 Hz, 1H), 4.80 (t, *J* = 4.8 and 46.8 Hz, 1H), 4.71 (t, *J* = 4.8 and 46.8 Hz, 1H), 4.33 (s, 2H), 2.50–2.47 (m, 2H), 1.48 (q, *J* = 7.5 Hz, 2H), 1.25 (h, *J* = 7.2 Hz, 2H), 0.81 (t, *J* = 7.3 Hz, 3H).^13^C NMR (75 MHz, CDCl_3_) δ_C_ 165.6, 148.7, 141.3, 140.8, 135.0, 130.8, 130.5, 130.4, 130.0, 128.0, 127.7, 126.1, 125.8, 124.9, 81.3, 79.0, 53.3, 53.0, 47.5, 29.9, 26.9, 22.6, 13.9. ^19^F NMR (282 MHz, CDCl_3_) δ_F_ −224.05 (referred to the TFA reference standard). HRMS (ES^+^): *m*/*z* [M + H]^+^ calculated for C_24_H_27_ClFN_6_O: 469.1919, found: 469.1923.

#### 4.2.3. Synthesis of 2-Butyl-4-chloro-5-(azidomethyl)-1-[(2′-(1*H*-tetrazol-5-yl)biphenyl-4-yl)methyl]-1*H*-imidazole (**7**)

The compound tetrazole-protected azido-losartan 6 was synthetized according to the literature [21]. Following a described procedure for deprotection of tetrazole-protected losartan [20], a solution of 1M HCl in methanol (1.7 mL) was added to a solution of 6 (15.7 mg, 22.8 µmol) in 1,4-dioxane (1.4 mL). After stirring at room temperature (22 °C) for 24 h, reaction was completed and monitored by HPLC (Phenomenex Luna C18 semi-preparative column (5 µ, 250 × 10 mm), 0–10 min, 60:40, 0.1% TFA water/MeCN, 11–15 min, 40–100% MeCN, 15–25 min, 100% MeCN, 4 mL/min, 254 nm; retention time of 16 min). The reaction was quenched, extracted with ethyl acetate and brine, dried over MgSO_4_, filtered and the solvent was evaporated. The residue was dissolved in MeCN and purified by HPLC (Phenomenex Luna C18 semi-preparative column (5 µ, 250 × 10 mm), 0–10 min, 60:40, 0.1% TFA water/MeCN, 11–15 min, 40–100% MeCN, 15–25 min, 100% MeCN, 4 mL/min, 254 nm; retention time of 16 min). The product was also purified by flash column chromatography (silica gel, hexane/ethyl acetate, 95:5–30:70). The collected fractions were pooled together, concentrated and the product was freeze-dried to get 9.8 mg (96%) of **7**. ^1^H NMR (300 MHz, CDCl_3_) δ_H_ 7.95 (dd, *J* = 7.5 Hz, 1.4 Hz, 1H), 7.66–7.51 (m, 2H), 7.42 (dd, *J* = 7.2 Hz, 1.5 Hz, 1H), 7.15 (d, *J* = 8.2 Hz, 2H), 6.85 (d, *J* = 8.1 Hz, 2H), 5.14 (s, 2H), 4.12 (s, 2H), 2.43 (t, 2H), 1.59 (p, *J* = 7.6 Hz, 2H), 1.32 (h, *J* = 7.4 Hz, 2H), 0.88 (t, *J* = 7.3 Hz, 3H). ^13^C NMR (75 MHz, CDCl_3_) δ_C_ 149.3, 140.6, 139.9, 135.1, 131.4, 131.1, 130.7, 130.0, 128.6, 120.3, 77.2, 47.3, 42.9, 30.0, 26.8, 22.5, 13.9. HRMS (ES^+^): *m*/*z* [M + H]^+^ calculated for C_22_H_23_ClN_9_: 448.1765; found: 448.1758.

#### 4.2.4. Synthesis of 2-Butyl-4-chloro-5-[((1*H*-1,2,3-triazol-4-yl)-(*N*,*N*-dimethyl-ammoniomethyl-trifluoroborate)methyl)methyl]-1-[(2′-(1*H*-tetrazol-5-yl)biphenyl-4-yl)methyl]-1*H*-imidazole (AMBF_3_Los)

AMBF_3_-alkynyl (**8**) was synthetized according to the literature [34,36]. Then, CuSO_4_ (1M, 15 µL), Na asc (1M, 40 µL), NH_4_OH 5% (vol/vol) (1:1 MeCN/H_2_O, 150 µL), compound **8** (6.6 mg, 40 µmol, 300 µL MeCN/H_2_O 1:1), and compound **7** (4.8 mg, 10.7 µmol, 700 µL of MeCN), were sequentially added to a 5 mL tube. After stirring at 45 °C for 2 h, the product was purified by HPLC (Phenomenex Luna C18 semi-preparative column (5 µ, 250 × 10 mm), 0–10 min, 80:20, Milli-Q water/MeCN, 11–25 min, 60:40 Milli-Q water/MeCN, 4 mL/min, 254 nm; retention time of 15.2 min). The collected fractions were frozen, and the product was freeze-dried to get 3.4 mg (52%) of AMBF_3_Los (Scheme 2). Then, the pure AMBF_3_Los was diluted in acetone and stored in aliquots of ~25 or 100 nmol for further radiolabeling; the solvent was blow-dried. ^1^H NMR (500 MHz, CD_3_CN) δ_H_ 7.96 (s, 1H), 7.80 (d, *J* = 7.4 Hz, 1H), 7.65 (t, *J* = 7.4 Hz, 1H), 7.56 (t, *J* = 7.4 Hz, 1H), 7.47 (d, *J* = 7.6 Hz, 1H), 6.96 (d, *J* = 8.1 Hz, 2H), 6.68 (d, *J* = 7.9 Hz, 2H), 5.49 (s, 2H), 5.45 (s, 1H), 5.24 (s, 2H), 4.28 (s, 2H), 3.27 (s, 1H), 2.83 (s, 6H), 2.58 (t, *J* = 7.6 Hz, 2H), 1.60 (q, *J* = 7.7 Hz, 2H), 1.33 (h, 2H), 0.87 (t, *J* = 7.3 Hz, 3H). ^13^C NMR (75 MHz, CD_3_CN) δ_C_ 149.7, 136.7, 131.3, 131.0, 130.7, 129.3, 128.0, 127.5, 125.6, 59.7, 52.5, 47.0, 42.5, 29.3, 26.3, 21.9, 13.0. ^19^F NMR (470 MHz, CD_3_CN) δ_F_ −137.25–−137.75 (m). HRMS (ES^+^): *m*/*z* [M + H]^+^ calculated for C_28_H_34_BClF_3_N_10_: 613.2702; found: 613.2693.

### 4.3. Radiochemistry

#### 4.3.1. Concentration and [^18^F]F^−^ Drying

The irradiated water containing the [^18^F]F^−^ was loaded onto a pre-conditioned QMA cartridge (Waters, Brazil) (130 mg, quaternary ammonium, carbonate form), and [^18^F]F^−^ was eluted off with 1 mL of K_2_CO_3_ (2 mg, 14 µmol)/Kryptofix 2.2.2 (K_222_, 11 mg, 29 µmol) mixture in 2:8 Milli-Q water/MeCN [29]. [K^+^/K_222_]/[^18^F]F^−^/CO_3_^2−^ complex was dried by azeotropic distillation at 100 °C and nitrogen atmosphere with sequential addition of MeCN (1 mL, 3 times).

#### 4.3.2. Radiosynthesis of 2-[^18^F]Fluoroethyl Tosylate (2*)

The solution of ethylene glycol bistosylate precursor (8 mg, 21.6 µmol) in anhydrous MeCN (1 mL) was added into the sealed conical reaction vial containing the dried [K^+^/K_222_]/[^18^F]F^−^/CO_3_^2−^ complex (1.6 ± 0.6 GBq = 44 ± 16 mCi). The reaction was set at 100 °C for one minute. Next, the crude mixture was injected onto a semi-prep HPLC system to purify the product (Phenomenex nucleosil C18 semi-preparative column (5 µm, 10 × 250 mm), 50:50, 0.1% TFA water/MeCN, 3 mL/min, 254 nm; retention time of 14 min). The collected fraction of 2* was diluted and loaded onto a Sep-Pak C18 Plus cartridge (Waters, Brazil). The loaded cartridge was washed with Milli-Q water (10 mL), and heated at 50 °C for 10 min while flushing with nitrogen [29]. Then, 2* was eluted off with DMF (1 mL). An aliquot of the final formulation was analyzed by HPLC (Agilent Zorbax Eclipse Plus C18 analytical column (5 µm, 4.6 × 250 mm), 0–15 min, 55:45, 0.1% TFA water/MeCN, 15–20 min, 45–100% MeCN, 20–30 min, 100% MeCN, 1 mL/min, 254 nm; retention time of 8.6 min) to confirm the identity of the labelled compound by a single peak on radiochromatogram compared to its cold compound on 254 nm UV profile, and evaluate the chemical and radiochemical purity.

#### 4.3.3. Radiosynthesis of [^18^F]FEtLos

A solution of 2* (0.20 ± 0.07 GBq = 5 ± 2 mCi) in DMF (800 μL) was dropwise added to the vial containing losartan potassium (1 mg, 2.2 µmol) and NaH (8 eq.) in DMF (30 μL) at room temperature. The reaction vial was heated at 80 °C for 20 min under stirring and nitrogen atmosphere (Scheme 3). The labeling reaction was monitored by analytical HPLC (Agilent Zorbax Eclipse Plus C18 analytical column (5 µm, 4.6 × 250 mm), 0–15 min, 55:45, 0.1% TFA water/MeCN, 15–20 min, 45–100% MeCN, 20–30 min, 100% MeCN, 1 mL/min, 254 nm; retention time of 7.9 min). The crude mixture was diluted with 5:5 MeCN/water mixture, and injected onto a semi-prep HPLC system (Phenomenex Nucleosil C18 semi-preparative column (5 µm, 10 × 250 mm), 55:45, 0.1% TFA water/MeCN, 3 mL/min, 254 nm; retention time of 21 min) to purify the [^18^F]FEtLos. The collected fraction was diluted with Milli-Q water and loaded onto a Sep-Pak C18 Plus cartridge (Waters, Brazil). Finally, [^18^F]FEtLos was eluted off with MeCN (1 mL), the solvent was evaporated at 100 °C, and the product was reformulated in saline for biological studies. An aliquot of the final formulation was analyzed by HPLC (Agilent Zorbax Eclipse Plus C18 analytical column (5 µm, 4.6 × 250 mm), 0–15 min, 55:45, 0.1% TFA water/MeCN, 15–20 min, 45–100% MeCN, 20–30 min, 100% MeCN, 1 mL/min, 254 nm; retention time of 7.9 min).

#### 4.3.4. Radiosynthesis of [^18^F]AMBF_3_Los

[^18^F]AMBF_3_Los was manually synthesized using both low (0.7 ± 0.2 GBq) or high (61 ± 23 GBq) activities of [^18^F]F^−^. Telemanipulators were used to handle the high activities of [^18^F]F^−^; whereas, low [^18^F]F^−^ activities were performed manually (without telemanipulators). Sodium fluoride was added as the source of carrier [^19^F]fluoride.

##### Radiosynthesis Using Low Activities (0.7 ± 0.2 GBq) of [^18^F]F^−^

AMBF_3_Los (25 nmol) was resuspended in a mixture of DMF (15 µL), aqueous pyridazine-HCl buffer (1.0 M, pH 2.0–2.5, 15 µL), and an aqueous solution of sodium fluoride (15 mM, 1 µL). No carrier-added [^18^F]F^−^ (0.7 ± 0.2 GBq; 0.019 ± 0.005 Ci), was trapped on the µ-QMA cartridge (ORTG Inc., Oakdale, TN, USA) (9 mg, quaternary ammonium, chloride form). The [^18^F]F^−^ was eluted with 100-µL of isotonic saline into a polypropylene tube (reaction vial) containing the precursor (AMBF_3_Los). The ^18^F-labeling reaction was allowed to react at 80 °C for 20 min under vacuum and quenched with NH_4_OH (5% in water, 2 mL). The reaction mixture was then loaded onto a Sep-Pak C18 light cartridge (Waters, Brazil), washed with 2-mL of Milli-Q water, and [^18^F]AMBF_3_Los (Scheme 4) was eluted with 0.5-mL of 1:1 ethanol/saline solution. The solvent was evaporated at 45 °C under a slow nitrogen flow, and the radiotracer was resuspended in saline for biological studies. An aliquot of the final formulation was analyzed by HPLC (Agilent analytical Zorbax Eclipse Plus C18 column (5 µm, 4.6 × 250 mm), solvent A: 0.1% TFA water, solvent B: MeCN, 0–30 min, 0–100% B, 1 mL/min, 254 nm; retention time of 17.6 min).

##### Radiosynthesis Using High Activities (61 ± 23 GBq) of [^18^F]F^−^

AMBF_3_Los (100 nmol) was resuspended in a mixture of DMF (15 µL), aqueous pyridazine-HCl buffer (1.0 M, pH 2.0–2.5, 15 µL), and an aqueous solution of sodium fluoride (15 mM, 1 µL). No carrier-added [^18^F]F^−^ (61 ± 23 GBq; 1.6 ± 0.6 Ci), was trapped on the µ-QMA cartridge (ORTG Inc., Oakdale, TN, USA) (9 mg, quaternary ammonium, chloride form). The [^18^F]F^−^ was eluted with 100-µL of an isotonic saline into a polypropylene tube (reaction vial) containing the precursor (AMBF_3_Los). The ^18^F-labeling reaction was allowed to react at 80 °C for 20 min under vacuum and quenched with NH_4_OH (5% in water, 2 mL). The reaction mixture was then loaded onto a Sep-Pak C18 light cartridge (Waters, Brazil), washed with 2-mL Milli-Q water, and [^18^F]AMBF_3_Los (Scheme 4) was eluted with 0.5-mL of 1:1 ethanol/saline solution. The tracer was then reformulated in saline (maximum of 10% ethanol solution) for biological studies. An aliquot of the final formulation was analyzed by HPLC (Agilent analytical Zorbax Eclipse Plus C18 column (5 µm, 4.6 × 250 mm), solvent A: 0.1% TFA water, solvent B: MeCN, 0–30 min, 0–100% B, 1 mL/min, 254 nm; retention time of 17.6 min).

#### 4.3.5. Quality Control

The chemical identity of [^18^F]FEtLos and [^18^F]AMBF_3_Los was determined by co-injection with their respective cold compounds onto HPLC using the analytical conditions previously described in each method of radiosynthesis. The radiochemical purity and molar activity of [^18^F]FEtLos and [^18^F]AMBF_3_Los were determined and the radiochemical yields were decay-corrected. The molar activity was always calculated at the end of each radiosynthesis using a calibration curve (nmol of the cold compound vs. area under the curve recorded from the HPLC analytical profile) (Figures S11 and S12).

##### Log D_7.4_ Measurements

The log D_7.4_ values were determined according to the literature [65]. Aliquots (2 μL) of [^18^F]FEtLos and [^18^F]AMBF_3_Los were added to polypropylene tubes containing 3 mL of octanol and 3 mL of PBS (phosphate-buffered saline). The samples were vortexed for 20 s (2 times) and centrifuged at 600× *g* for 15 min. Aliquots (0.1 mL) of each layer were counted in a gamma counter to determine the log D_7.4_ as log (counts in octanol layer/counts in PBS layer).

### 4.4. In Vitro Assays

#### 4.4.1. Competition Binding Assays in Membranes Containing Human AT_1_R

The assays were performed using membranes expressing the human angiotensin II type 1 receptor from CHO-K1 cells, purchased from Perkin Elmer (Waltham, MA, USA). A serial dilution ranging from 10^−4^ to 10^−12^ M of Losartan potassium, FEtLos and AMBF_3_Los were used as cold ligands to displace the hot [^125^I]-(Sar1,Ile8)-Angiotensin II (0.3 nM taking into account the activity of ^125^I decay-corrected at the day of the assay and the specific activity of the hot compound (81.4 TBq (2200 Ci)/mmol)). The hot ligand [^125^I]-(Sar1,Ile8)-Angiotensin II (81.4 TBq/mmol) was also purchased from Perkin Elmer (USA). The Multiscreen MSFCN6B50 96-well plates were used for this assay. The procedure was based on the recommended assay conditions from Perkin Elmer (Waltham, MA, USA) and procedures previously described for other AT_1_R ligands [22,23]. Briefly, the 96-well plate was preincubated for 1–2 h at 4 °C with 200 μL of 0.5% BSA (Bovine Serum Albumin) followed by washing (3 times) with an assay buffer. Next, 150 µL of the membranes, diluted (1:150) in the buffer assay (50 mM Tris-HCl pH 7.4, 5 mM MgCl_2_) to a final concentration of 0.6 μg/well, were added to the wells followed by the addition of cold (25 µL) and hot ligand (25 µL). The plate was incubated for 60 min at 27 °C under stirring and at the end of the experiment all wells were washed (9 times) with ice-cold wash buffer (50 mM Tris-HCl pH 7.4) using vacuum. Afterwards, membranes were punched out and counted in a Perkin Elmer (Waltham, MA, USA) Wizard2 2480 automatic gamma counter. The Ki values were calculated by a non-linear curve fitting using GraphPad Prism 7.01 Software (San Diego, CA, USA). The experiment was done in triplicate.

#### 4.4.2. Cell Culture

Chinese hamster ovary cells CHO and CHO-AT1R (a gift of Professor Dr. Claudio Costa-Neto from the Department of Biochemistry and Immunology, at the School of Medicine of Ribeirão Preto, University of São Paulo, Ribeirão Preto, Brazil), were cultured in high glucose Dulbecco’s modification of Eagle medium (DMEM) (Gibco, Life technologies, MD, USA), supplemented with 10% of fetal bovine serum (FBS) (Gibco, Life technologies, MD, USA), penicillin (100 U/mL) and streptomycin (100 µg/mL) (Sigma-Aldrich, St. Louis, MO, USA), and kept at 37 °C and 5% CO_2_.

#### 4.4.3. Real-Time Polymerase Chain Reaction (RT-PCR)

The AT1R mRNA abundance was measured in CHO-AT1R and CHO cells by RT-PCR. Total RNA from cells was isolated with 1 mL of Tri-Reagent (Sigma-Aldrich, St. Louis, MO, USA) according to the manufacturer’s instructions. Complementary DNA was synthesized using the High capacity cDNA RT kit (Applied Biosystems, Foster City, CA, USA), according to the manufacturer’s protocols. Quantitative PCR analysis was performed in triplicate using the Power SYBR^®^ Green PCR Master Mix (Applied Biosystems). Relative quantification was done using the ΔΔCt method normalizing to β-actin gene expression. Primer human AT_1_R: forward 5′–3′, GCGTCAGTTTCAACCTC; reverse 5′–3′, TCCGGGACTCGTAATG. Primer hamster β-actin: Forward 5′–3′, GGCAGGCAAAGGTTACTCTG; reverse 5′–3′, TGGTGACAGGTGGACAAGAT.

#### 4.4.4. Cell Binding Assays

The cell binding assays were performed based on previous reports [66] with modifications. CHO-AT1R and CHO cells (2 × 10^5^) were plated in a 6-well plate overnight and then incubated for one hour at 4 °C with 10 µCi (370 kBq) of [^18^F]AMBF_3_Los per well, in presence or absence of the AT_1_R blocker (losartan potassium (100 µM/well) in PBS). To saturate the AT_1_R, the cells of the blocking group were treated with losartan 30 min before adding the radiolabelled compound. At the end of the incubation period, the supernatant was aspirated and cells were washed six times with ice-cold PBS, and further removed with cell scraper and transferred to a gamma counter tube. The cellular activity was counted in a Cobra II gamma counter (Packard).

### 4.5. In Vivo Assays

#### 4.5.1. Animals

Experiments involving animals were approved by the Animal Ethics Committee of the University of British Columbia (Protocol number: A16-0128). Immunodeficient NOD.Cg-*Prkdc*^scid^II2rg^tm1Wjl^/SzJ (NSG) mice were obtained from an in-house breeding colony at the Animal Resource Centre at BC Cancer Research Facility. Mice were always divided into baseline and AT_1_R blocked groups to assess the AT_1_R binding specificity of [^18^F]AMBF_3_Los with the co-injection of the AT_1_R blocker losartan (18 mg/kg, dissolved in PBS).

#### 4.5.2. Imaging and Biodistribution Studies

The in vivo AT_1_R binding specificity of [^18^F]AMBF_3_Los was evaluated in healthy mice by a dynamic PET scan. Healthy NSG mice were injected in the tail vein with 6 ± 1 MBq (170 ± 38 μCi) of [^18^F]AMBF_3_Los and a sixty-minute dynamic PET scans was carried out on a μPET/CT scanner (Inveon, Siemens) following procedures reported previously [67]. Alternatively, [^18^F]AMBF_3_Los was concomitantly injected with the AT_1_R blocker losartan (18 mg/kg) before the dynamic acquisition. Before and during the acquisition, mice were sedated with a continuous flow of isoflurane anesthesia (2% isoflurane in oxygen). The µPET/CT images were analyzed with PMOD v3.3 software (PMOD Technologies, Zürich, Switzerland). An elliptical volume-of-interest that enclosed the entire kidney was positioned manually on the PET images for the determination of the kidney volume. Then, 3-dimensional isocontours were drawn automatically. For each VOI, the percentage of the injected dose in the region of interest, divided by the injected dose per animal weight (% ID/g) could be calculated directly using the output parameters from the μPET.

After the sixty-minute dynamic PET scans, anesthetized mice were euthanized by carbon dioxide asphyxiation and blood, fat, testis, intestines, stomach, spleen, liver, pancreas, adrenal glands, kidneys, lungs, heart, muscle, bone and brain were harvested, rinsed in PBS, weighted and counted in a Perkin Elmer (Waltham, MA, USA) Wizard2 2480 automatic gamma counter to quantify the percent of injected dose per gram of tissue (% ID/g).

#### 4.5.3. Autoradiography

Kidneys harvested from healthy NSG mice (after the biodistribution studies), were rinsed in PBS and frozen in isopentane/dry-ice bath. Ten μm-thick slides of the kidneys were obtained using a cryostat (Leica, Wetzlar, Germany), and thaw-mounted onto Superfrost Plus microscope slides (Fischerbrand from Thermo Fisher Scientific, Toronto, ON, Canada), following general procedures of the lab [67]. Afterwards, kidney slides were exposed to a phosphor screen overnight and imaged on a Typhoon FLA 9500 scanner (GE Healthcare, Chicago, IL, USA).

#### 4.5.4. Statistical Analysis

Data was expressed as the mean ± standard deviation (SD). The statistical analysis was performed using GraphPad Prism 7.01 Software (San Diego, CA, USA). Data was analyzed by one unpaired *t*-test (multiple *t* tests). The outliers were removed before analyzing the data. A *p*-value < 0.05 was considered statistically significant.

## 5. Conclusions

Both FEtLos and AMBF_3_Los were successfully synthesized as novel derivatives of losartan. The radiolabelled derivative [^18^F]FEtLos was synthetized high radiochemical purity and low molar activity due to the small amount of ^18^F used, and resulted in a less lipophilic compound than losartan. Still, [^18^F]FEtLos exhibited a low AT_1_R binding affinity. On the other hand, [^18^F]AMBF_3_Los was synthetized with a high radiochemical purity and molar activity. Moreover, [^18^F]AMBF_3_Los was slightly hydrophilic, and showed a high AT_1_R binding affinity and specificity. Our data suggests that [^18^F]AMBF_3_Los might be a valuable PET imaging tracer for monitoring AT_1_R expression in several diseases.

## Figures and Tables

**Table 1:** Biodistribution of [^18^F]AMBF_3_Los in healthy NOD.Cg-Prkdc^scid^Il2rg^tm1Wjl^/SzJ mice.

Tissues	Baseline	AT_1_R Blocking
Blood	0.05 ± 0.03	0.08 ± 0.08
Fat	0.011 ± 0.004	0.008 ± 0.002
Testis	0.03 ± 0.01	0.029 ± 0.008
Intestines	63 ± 9	62 ± 3
Stomach	0.06 ± 0.05	2 ± 2
Spleen	0.2 ± 0.1	0.08 ± 0.03
Liver	0.5 ± 0.5	0.6 ± 0.3
Pancreas	0.05 ± 0.03	0.07 ± 0.06
Adrenal glands	0.1 ± 0.1	0.11 ± 0.06
Kidneys	3.5 ± 1.2	1.9 ± 1.3
Lungs	0.06 ± 0.03	0.08 ± 0.04
Heart	0.03 ± 0.01	0.04 ± 0.03
Muscle	0.02 ± 0.01	0.05 ± 0.04
Bone	0.11 ± 0.06	0.11 ± 0.03
Brain	0.006 ± 0.005	0.005 ± 0.004

Uptake data are presented as mean (% ID/g of tissues) ± SD of three independent experiments (*n* = 3).

## Supplementary Materials

The following are available online. Figure S1: ^1^H-NMR, ^13^C-NMR, ^19^F-NMR, and HRMS (ES^+^) spectra of compound **5**. Figure S2: ^1^H-NMR, ^13^C-NMR, ^19^F-NMR, HMBC and HRMS (ES^+^) spectra of compound FEtLos. Figure S3: ^1^H-NMR, ^13^C-NMR, ^13^F-NMR, HMBC and HRMS (ES^+^) spectra of compound **3**. Figure S4: ^1^H-NMR, ^13^C-NMR and HRMS (ES^+^) spectra of compound **7**. Figure S5: ^1^H NMR, ^13^C-NMR, ^19^F-NMR, HMBC, HMBC and HRMS (ES^+^) spectra of compound AMBF_3_Los. Figure S6: Quality control of the ^18^F-labeled compounds. Figure S7: In vitro assays with [^18^F]FEtLos. Figure S8. Ex vivo assays with [^18^F]FEtLos. Figure S9: Structure of NO-releasing derivatives of losartan. Figure S10: Structure of chelate-coupled losartan-Leucine-Diglycoloyl-Tetraethyleneglycol–Tetraamine. Figure S11: Calibration curve for determining the molar activity of [^18^F]AMBF_3_Los. Figure S12: Calibration curve for determining the molar activity of [^18^F]FEtLos. Supplemental material and methods.
