# Supplementary material for: Synthesis and Evaluation of [18F]FEtLos and [18F]AMBF3Los as Novel 18F-Labelled Losartan Derivatives for Molecular Imaging of Angiotensin II Type 1 Receptors
Source: Molecules. 2020 Apr 18;25(8):1872. doi: 10.3390/molecules25081872 (PMC7221519; doi:10.3390/molecules25081872)
Supplement: Supplementary file 1 [file molecules-25-01872-s001.pdf]

Supplementary Information

Synthesis and evaluation of [^{18}F]FEtLos and [^{18}F]AMBF$_3$Los as novel ^{18}F-labelled losartan derivatives for molecular imaging of angiotensin II type 1 receptors

Martha Sahylí Ortega Pijeira [1], Paulo Sérgio Gonçalves Nunes[2], Sofia Nascimento dos Santos[1], Zhengxing Zhang[3], Arian Pérez Nario[1], Efrain Araujo Perini[1], Walter Turato[4], Zalua Rodríguez Riera[5], Roger Chammas[6], Philip H. Elsinga[7], Kuo-Shyan Lin[3], Ivone Carvalho[2], and Emerson Soares Bernardes[1,*]

[1] Radiopharmacy Center, Nuclear and Energy Research Institute (IPEN / CNEN - SP), CEP 05508-000 São Paulo, SP, Brazil; msopijeira2015@usp.br , snsantos@usp.br , apereznario@usp.br , eaperini@ipen.br , ebernardes@ipen.br
[2] School of Pharmaceutical Sciences of Ribeirão Preto, University of São Paulo (FCFRP – USP), CEP 14040-903, Ribeirão Preto, Brazil; paulosgn@fcfrp.usp.br , carronal@usp.br
[3] Department of Molecular Oncology, BC Cancer Research Centre, Vancouver, BC V5Z 1L3, Canada; klin@bccrc.ca , zzhang@bccrc.ca
[4] School of Pharmaceutical Sciences – University of São Paulo - USP, CEP 05508-000, São Paulo, Brazil; turato@usp.br
[5] Departamento de Radioquímica, Instituto Superior de Tecnologías y Ciencias Aplicadas (InSTEC), Universidad de La Habana, CP 10400, La Habana, Cuba; zalua@instec.cu
[6] Department of Radiology and Oncology, Faculty of Medicine, University of São Paulo, CEP: 01246903, São Paulo, Brazil; rchammas@usp.br
[7] Department of Nuclear Medicine and Molecular Imaging, University Medical Center Groningen, University of Groningen, Internal Post code: EB79, PO box 30.001, 9700 RB, Netherlands; p.h.elsinga@umcg.nl
* Correspondence: ebernardes@ipen.br; Tel.: +55-11-3133-9529

Received: 16 December 2019; Accepted: date; Published: date

CONTENT

Figure S1. ^{1}H NMR, ^{13}C NMR, ^{19}F NMR, and HRMS (ES^{+}) spectra of compound **5**	3
Figure S2. ^{1}H NMR, ^{13}C NMR, ^{19}F NMR, HMBC and HRMS (ES^{+}) spectra of compound **FEtLos**	5
Figure S3. ^{1}H NMR, ^{13}C NMR, ^{13}F NMR, HMBC and HRMS (ES^{+}) spectra of compound **3**	9
Figure S4. ^{1}H NMR, ^{13}C NMR and HRMS (ES^{+}) spectra of compound **7**	12
Figure S5. ^{1}H NMR, ^{13}C NMR, ^{19}F NMR, HMQC, HMBC and HRMS (ES^{+}) spectra of compound **AMBF$_3$Los**	14
Figure S6. Quality control of the ^{18}F-labeled compounds	18
Figure S7. In vitro assays with **[^{18}F]FEtLos**	19
Figure S8. Ex vivo assays with **[^{18}F]FEtLos**	19
Figure S9. Structure of NO-releasing derivatives of losartan	20
Figure S10. Structure of chelate-coupled losartan-Leucine-Diglycoloyl-Tetraethyleneglycol-Tetraamine	20
Figure S11. Calibration curve for determining the molar activity of [^{18}F]AMBF$_3$Los.	20
Figure S12. Calibration curve for determining the molar activity of [^{18}F]FEtLos.	21
Supplemental material and methods	21
References	22

Figure S1.

^1H NMR (300 MHz, $CDCl_3$) spectrum of 2-butyl-4-chloro-5-(2-fluoroethoxy)methyl-1-[(2′-(1*H*-(1-(triphenylmethyl))tetrazol-5-yl)biphenyl-4-yl)methyl]-1*H*-imidazole (**5**)

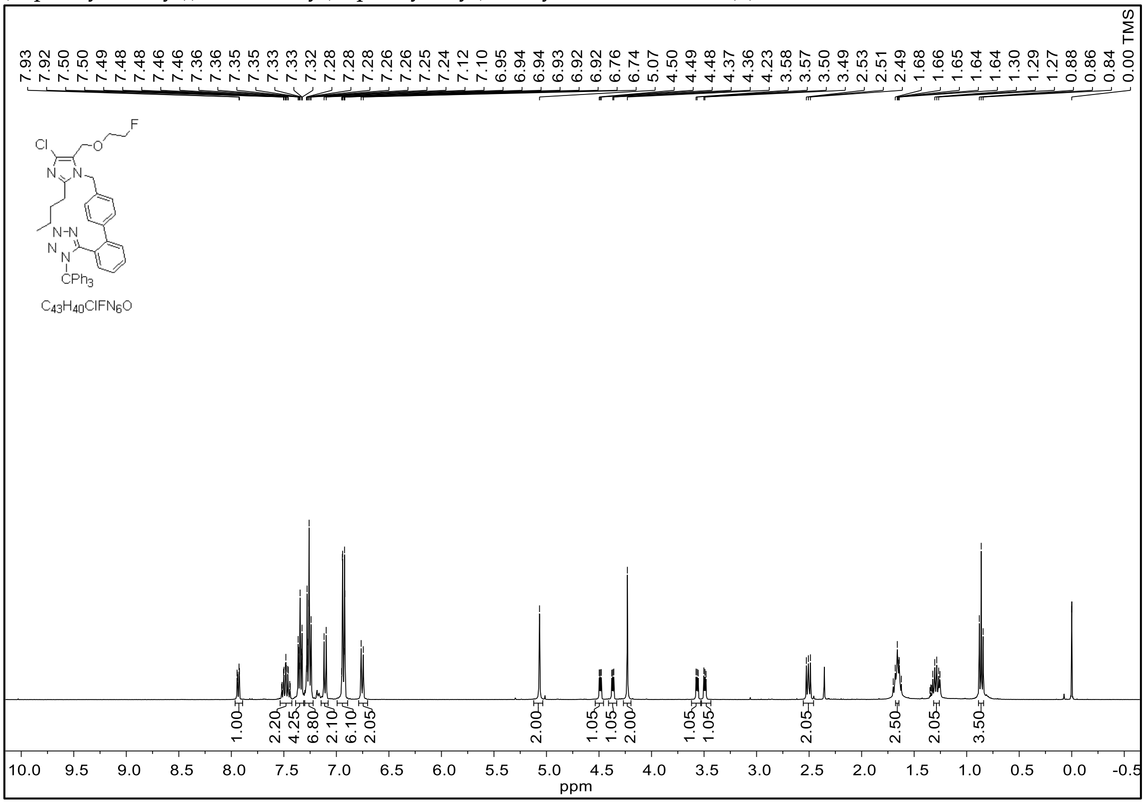

^{13}C NMR (75 MHz, $CDCl_3$) spectrum of **5**.

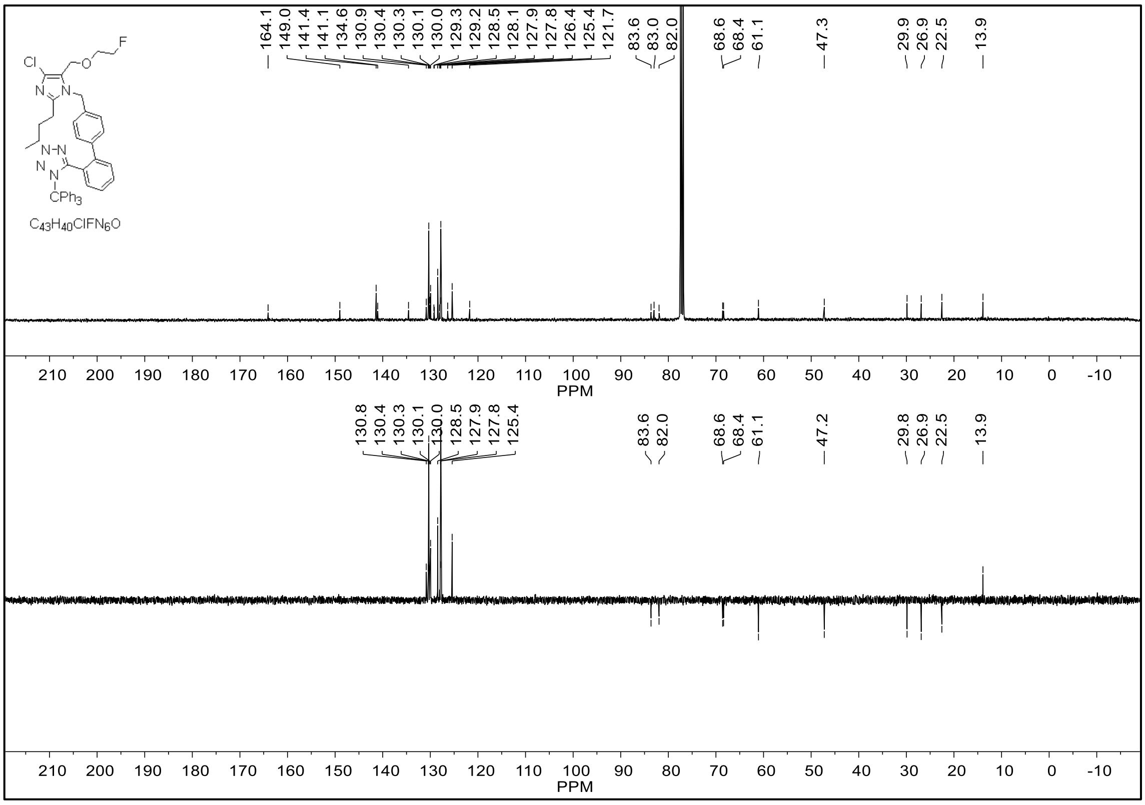

^{19}F NMR (282 MHz, $CDCl_3$; TFA reference standard) spectrum of **5**.

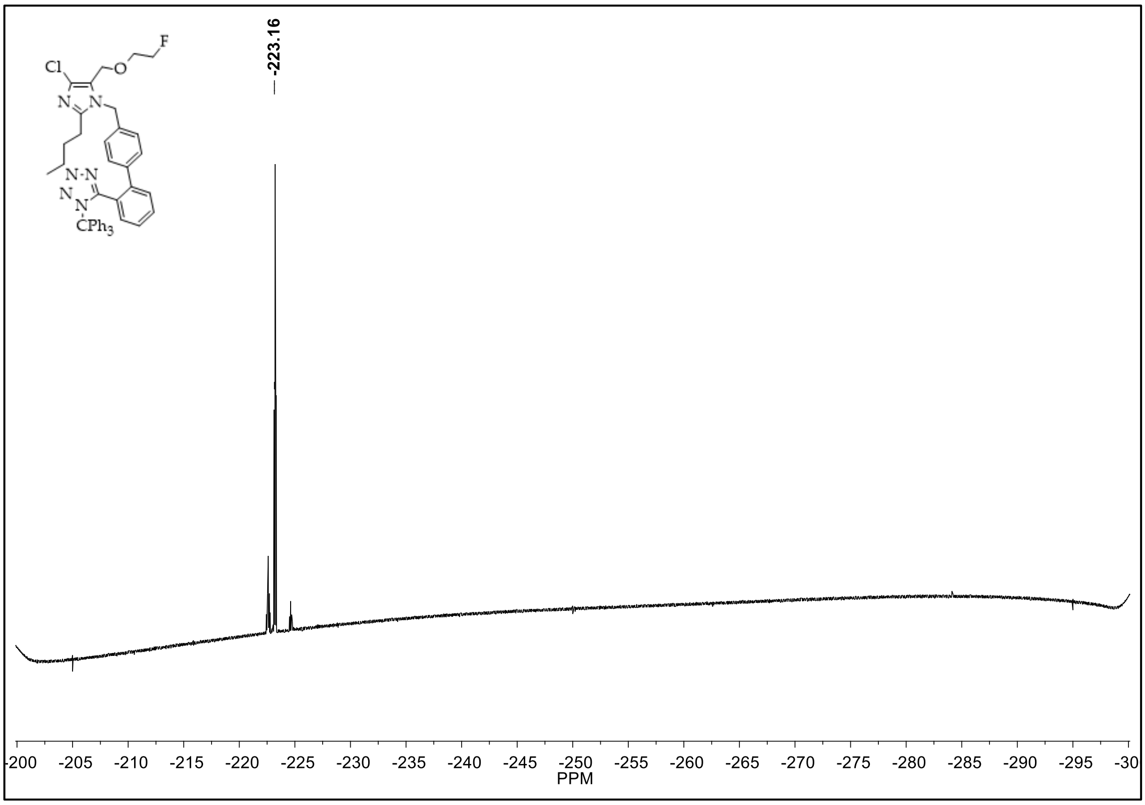

HRMS (ES$^+$): *m/z* $[M+H]^+$ calculated for $C_{43}H_{41}ClFN_6O$: 711.3014; found: 711.3018 (**5**).

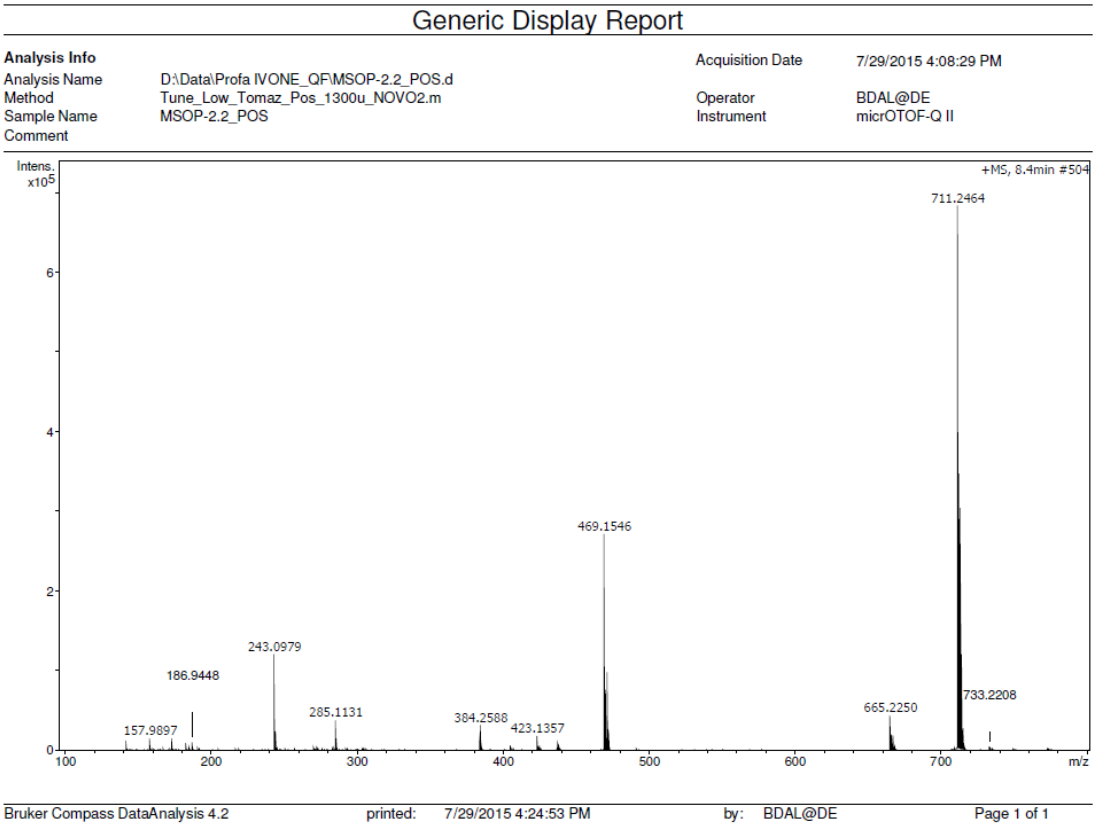

Figure S2.
^{1}H NMR (500 MHz, $CDCl_3$) spectrum of 2-butyl-4-chloro-5-(2-fluoroethoxy)methyl-1-[(2′-(1*H*-tetrazol-5-yl)biphenyl-4-yl)methyl]-1*H*-imidazole (**FEtLos**).

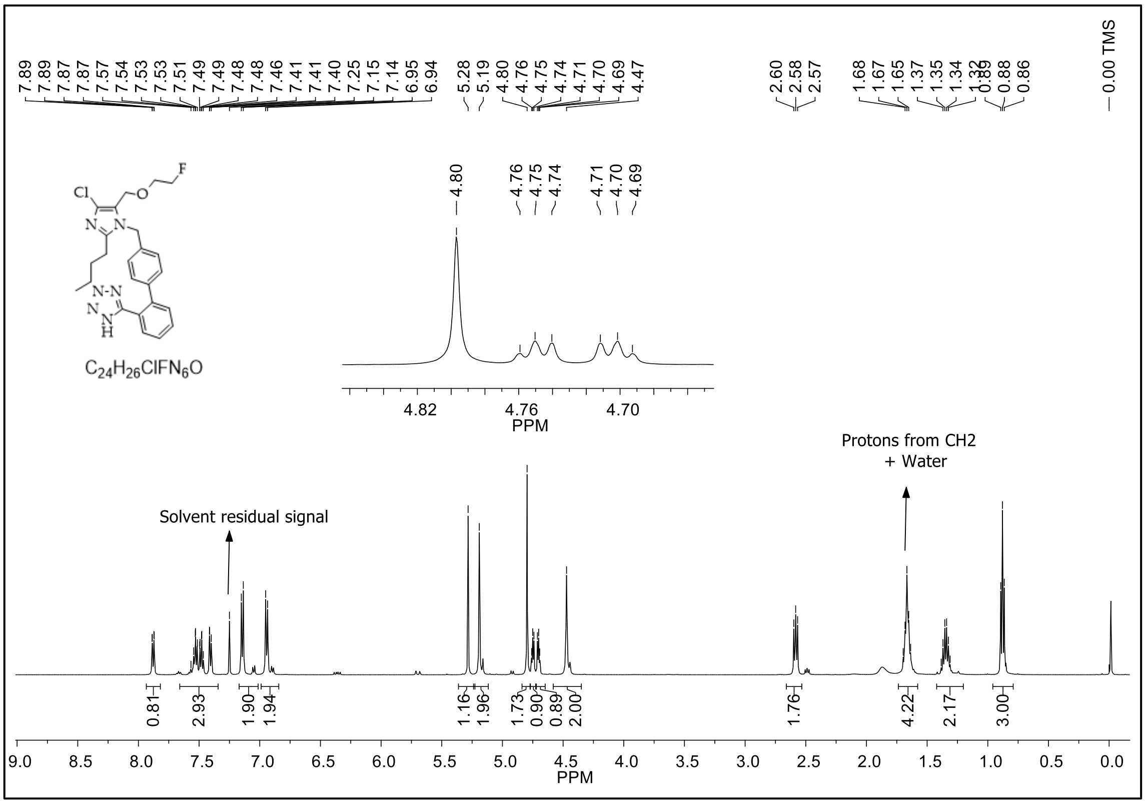

^{1}H NMR (500 MHz, DMSO-d6) spectrum of **FEtLos**.

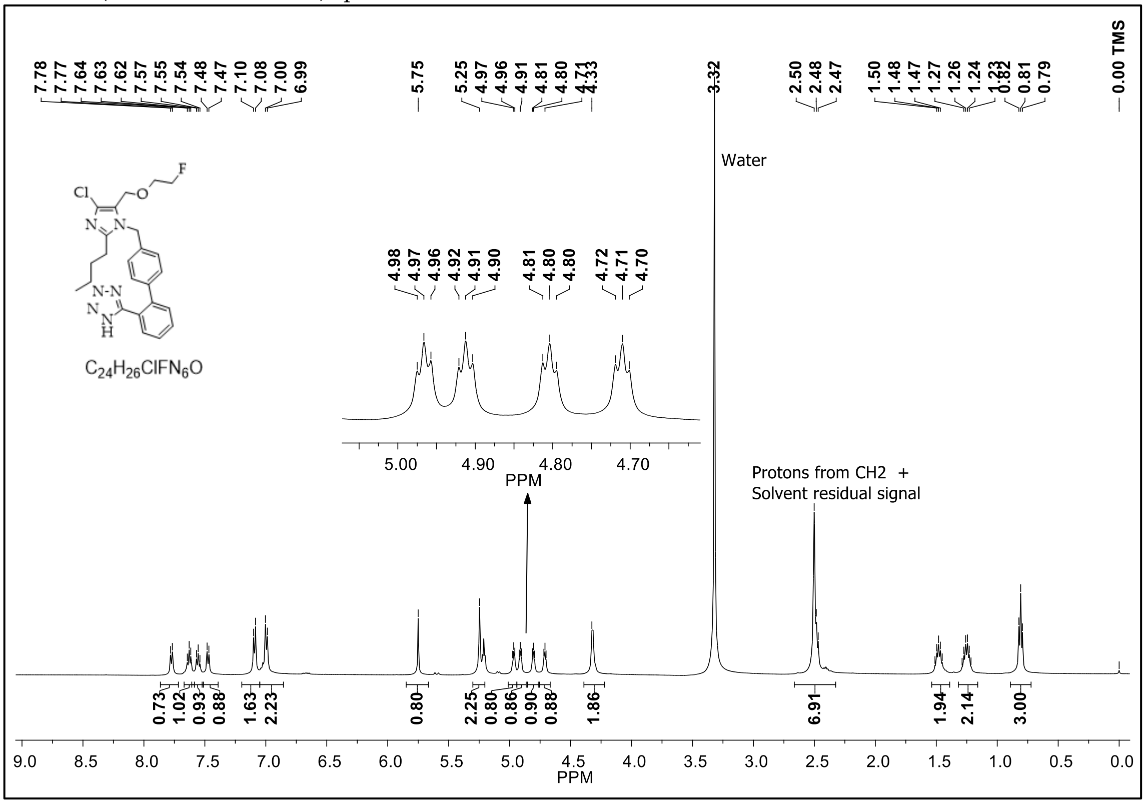

^{13}C NMR (75 MHz, CDCl3) spectrum of **FEtLos**.

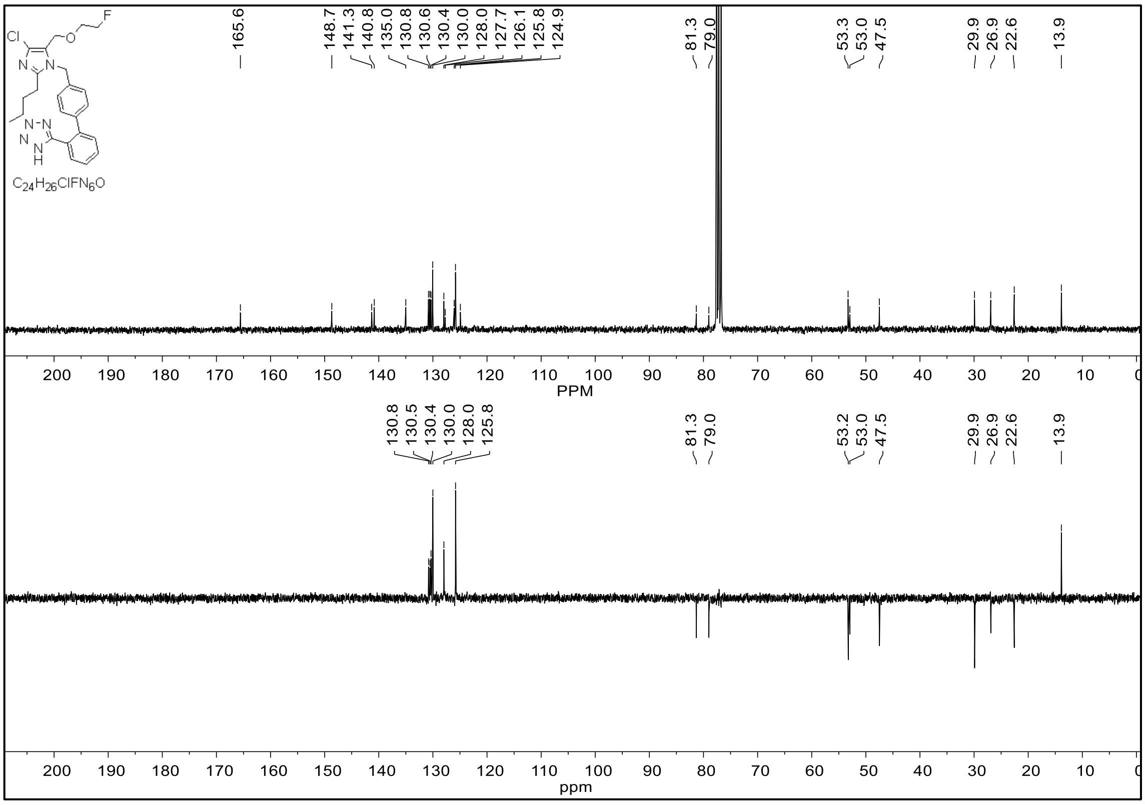

^{19}F NMR (282 MHz, $CDCl_3$; TFA reference standard) spectrum of **FEtLos**.

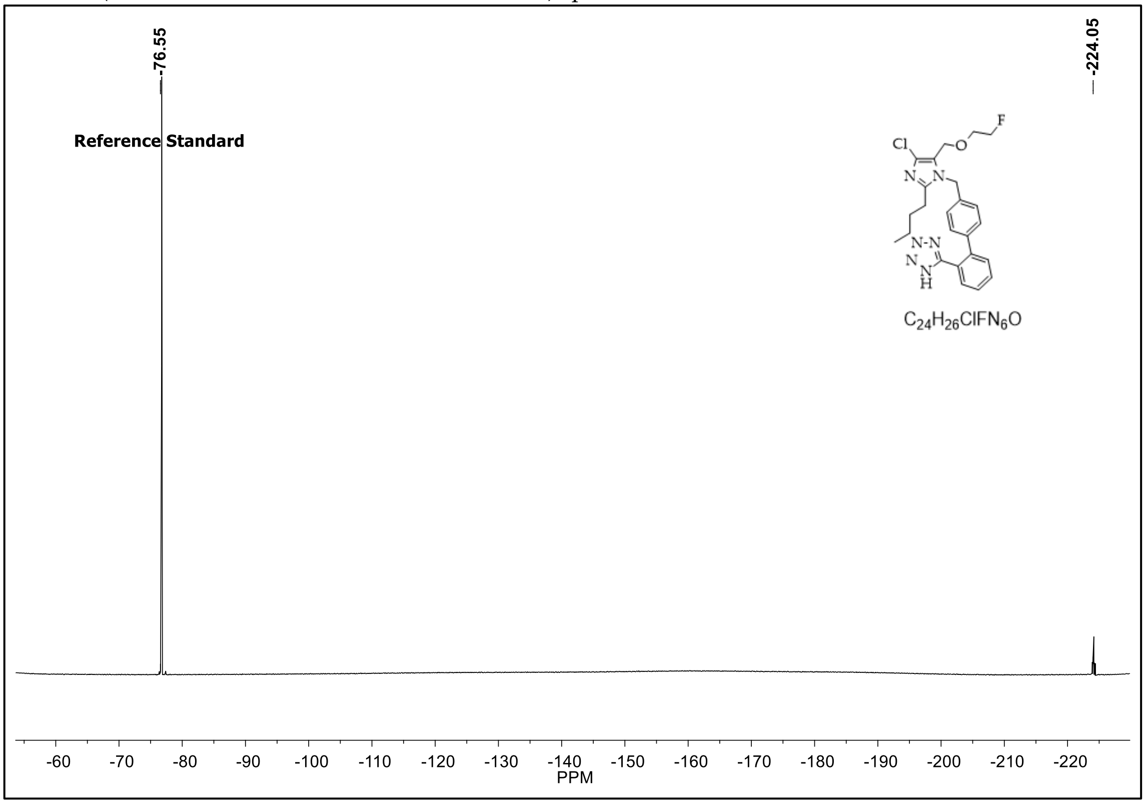

HMBC (300 MHz, $CDCl_3$) spectrum of **FEtLos**.

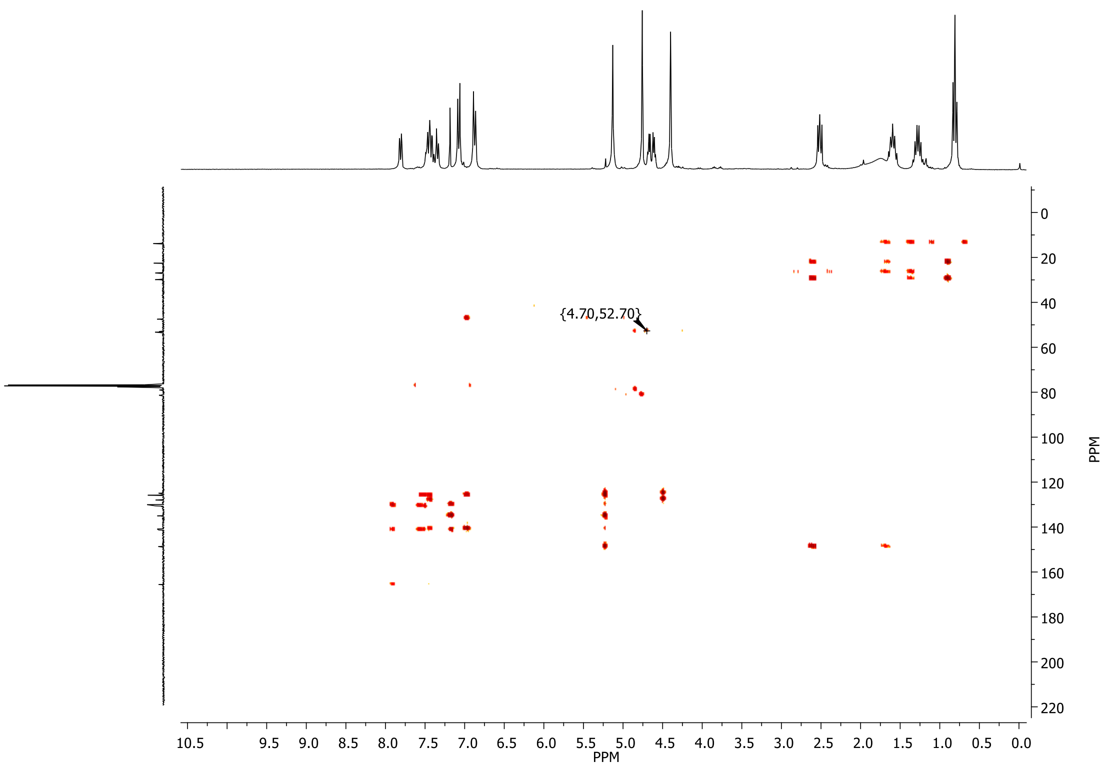
{4.70,52.70}
0
20
40
60
80
100
120
140
160
180
200
220
PPM
10.5
9.5
9.0
8.5
8.0
7.5
7.0
6.5
6.0
5.5
5.0
4.5
4.0
3.5
3.0
2.5
2.0
1.5
1.0
0.5
0.0
PPM

HRMS (ES+): *m/z* $[M+H]^+$ calculated for $C_{24}H_{27}ClFN_6O$: 469.1919, found: 469.1923 (**FEtLos**).

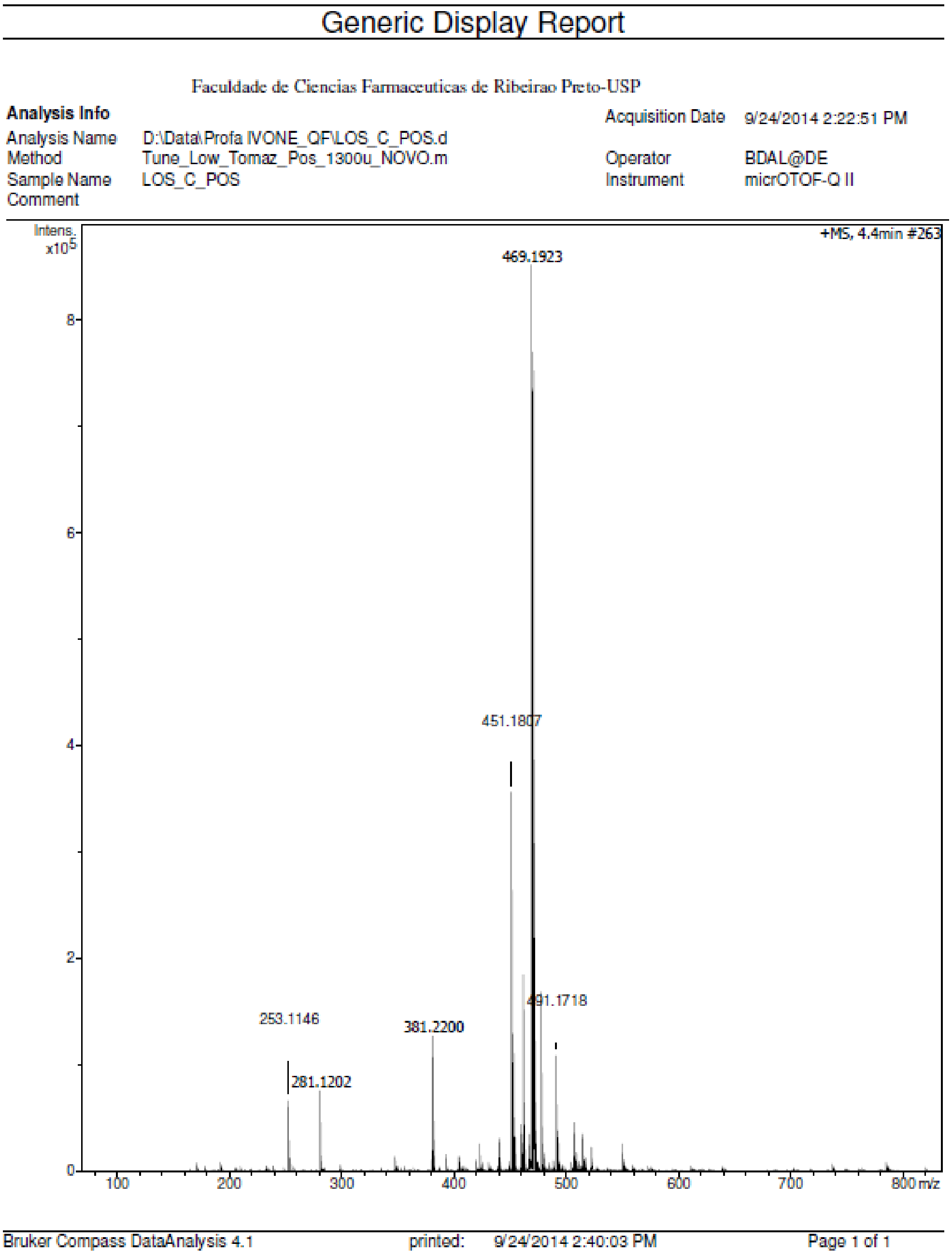

Figure S3.

^{1}H NMR (300 MHz, $CDCl_3$) spectrum of 2-butyl-4-chloro-5-(hydroxymethyl)-1-[(2′-(1*H*-(1-(2-fluoroethyl))tetrazol-5-yl)biphenyl-4-yl)methyl]-1*H*-imidazole (**3**).

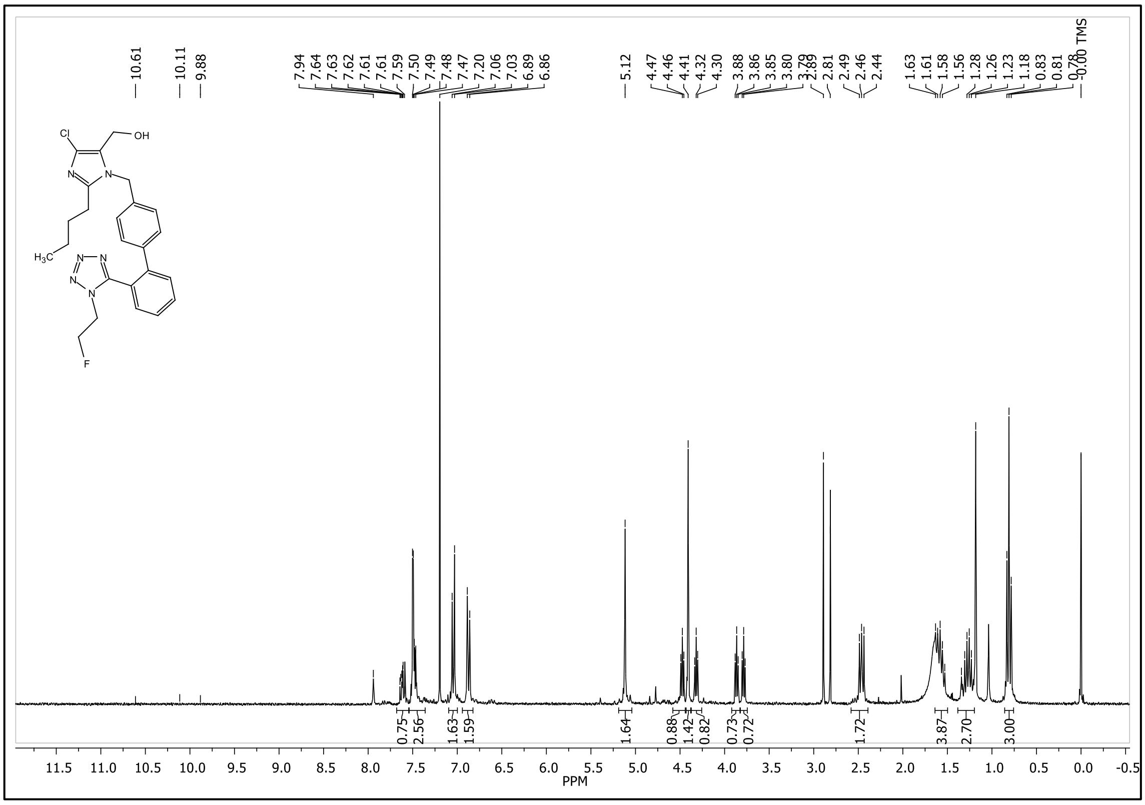

^{13}C NMR (75 MHz, $CDCl_3$) spectrum of **3**.

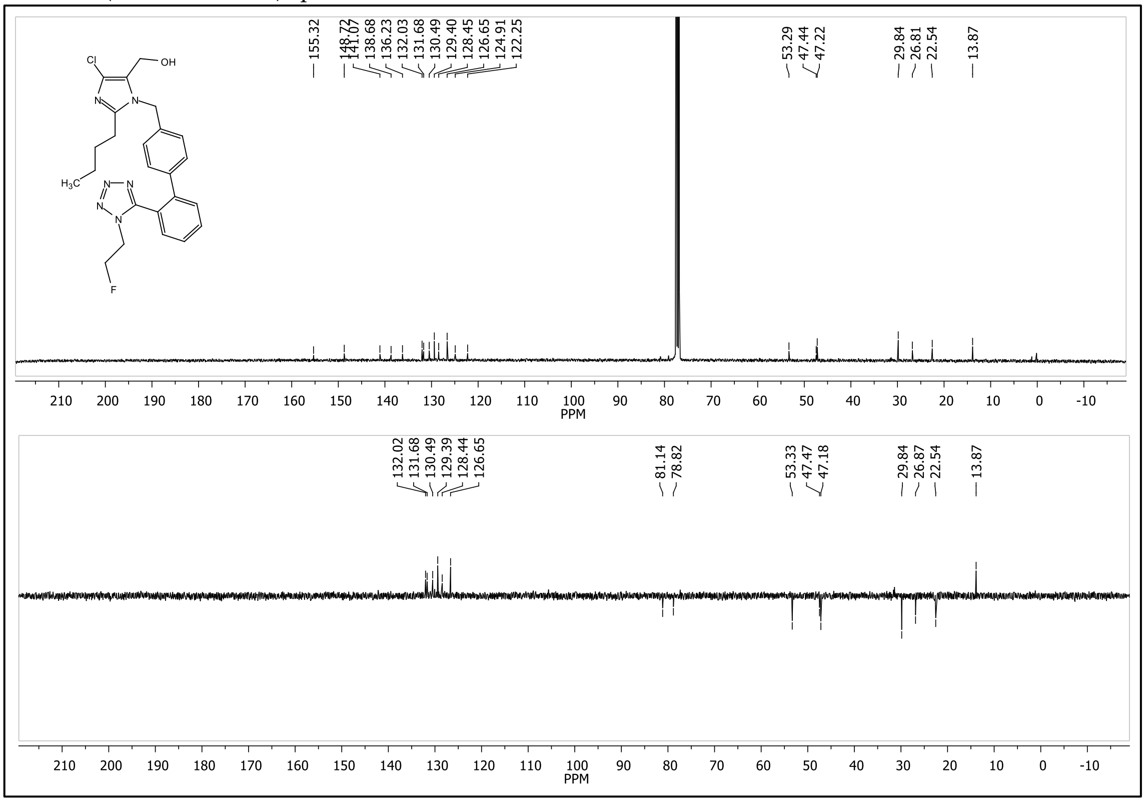

^{19}F NMR (282 MHz, $CDCl_3$) spectrum of **3**. The spectrum is referring to the TFA reference standard at 0 ppm instead of -76.55 ppm

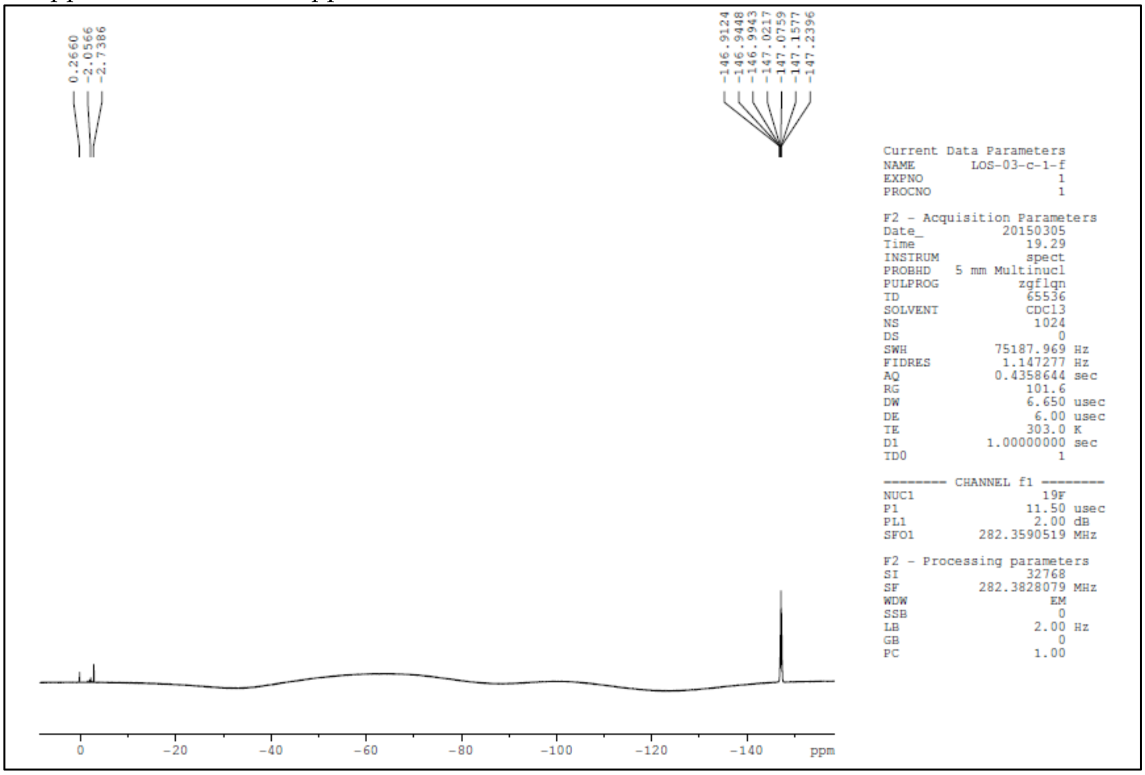

HMBC (300 MHz, $CDCl_3$) spectrum of **3**.

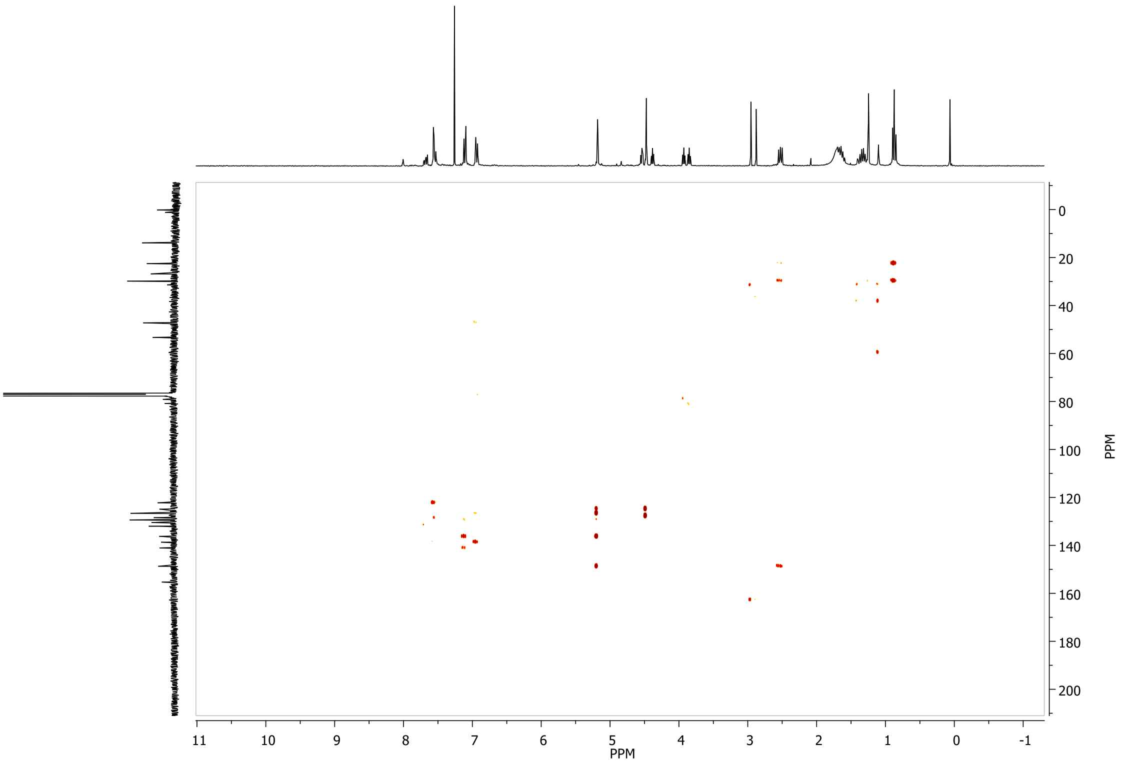

HRMS (ES$^+$): *m/z* [M+H]$^+$ calculated for $C_{24}H_{27}ClFN_6O$: 469.1919, found: 469.1919 (**3**).

Generic Display Report

Faculdade de Ciencias Farmaceuticas de Ribeirao Preto-USP

Analysis Info

Analysis Name	D:\Data\Profa IVONE_QF\LOS_C 1_POS.d	Acquisition Date	9/24/2014 2:09:17 PM
Method	Tune_Low_Tomaz_Pos_1300u_NOVO.m	Operator	BDAL@DE
Sample Name	LOS_C 1_POS	Instrument	micrOTOF-Q II
Comment			

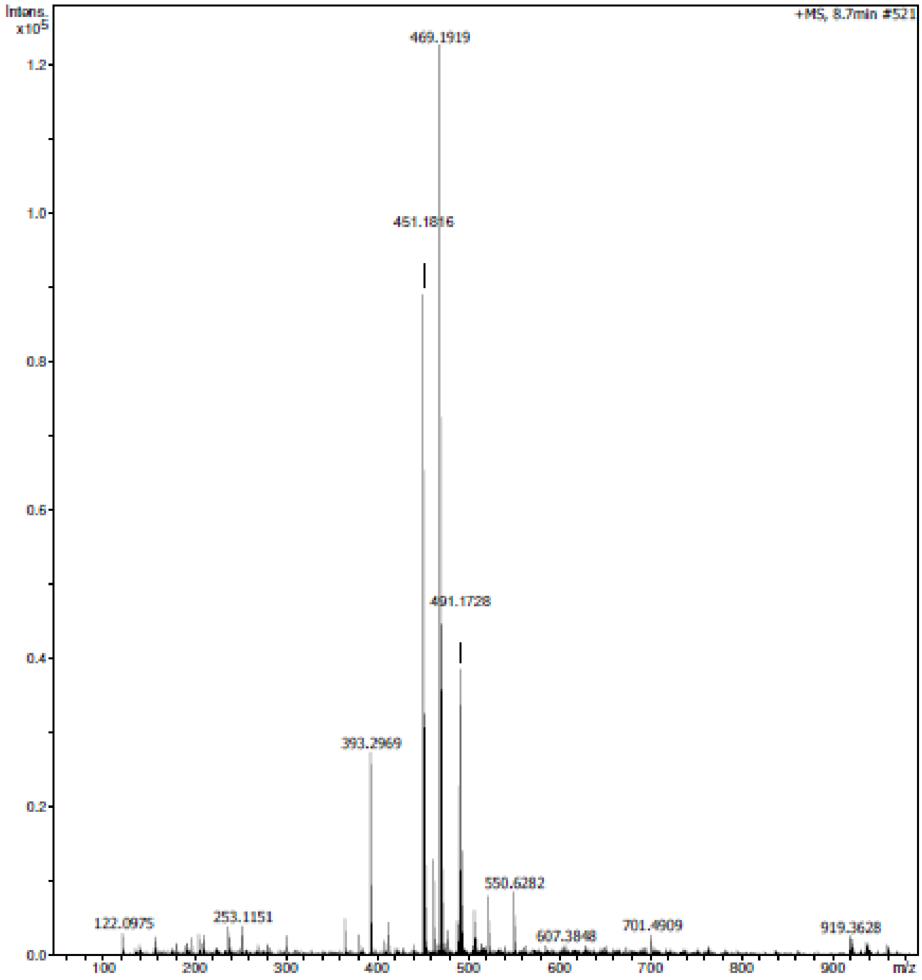

Bruker Compass DataAnalysis 4.1 printed: 9/24/2014 2:21:29 PM Page 1 of 1

Figure S4.

^{1}H NMR (300 MHz, $CDCl_3$) spectrum of 2-butyl-4-chloro-5-(azidomethyl)-1-[(2′-(1*H*-tetrazol-5-yl)biphenyl-4-yl)methyl]-1*H* -imidazole (**7**).

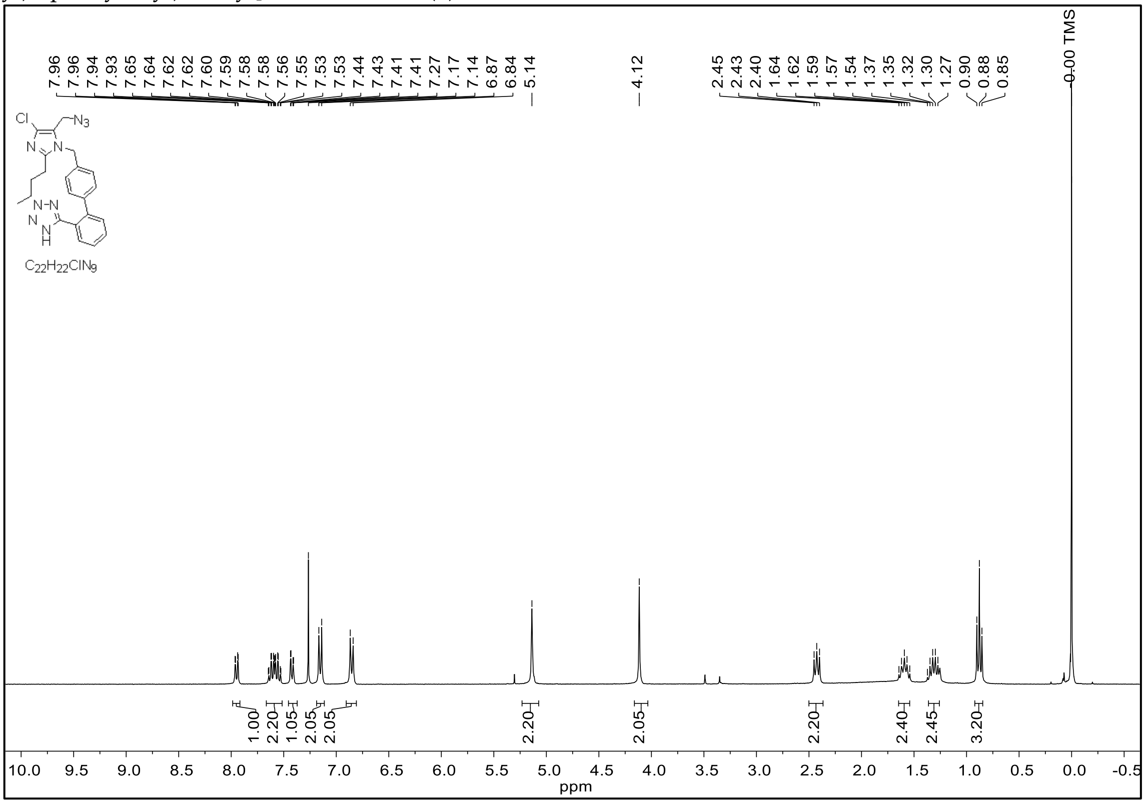

^{13}C NMR (75 MHz, $CDCl_3$) spectrum of **7**.

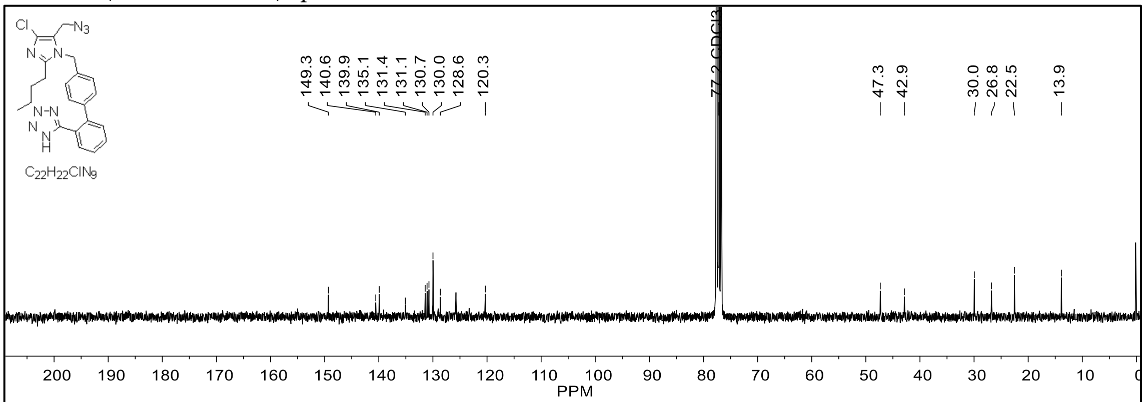

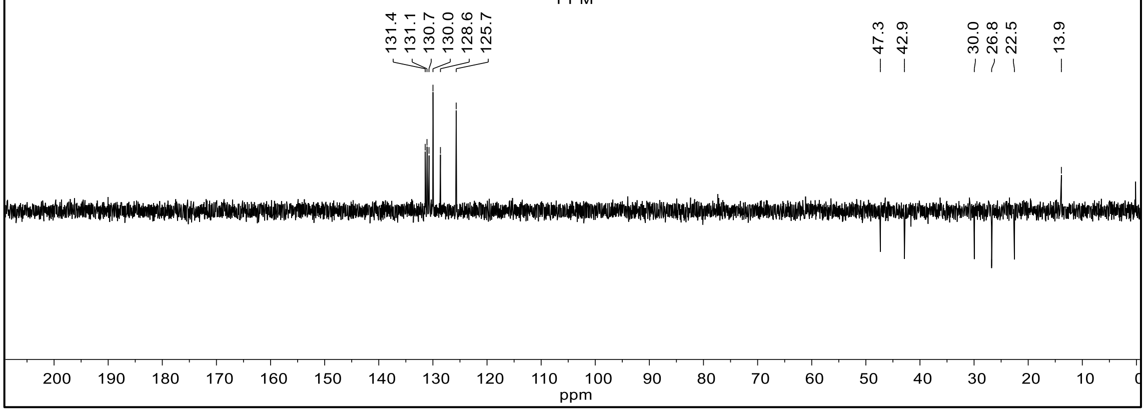

HRMS (ES+): m/z $[M+H]^+$ calculated for $C_{22}H_{23}ClN_9$: 448.1765; found: 448.1758 (**7**).

Generic Display Report

Analysis Info

Analysis Name	D:\Data\PROF. IVONE\N 3_Los_POS.d	Acquisition Date	12/3/2018 9:40:11 AM
Method	Tune_Low_Tomaz_Pos_1300u_Willian 1.m	Operator	TOMAZ
Sample Name	N 3_Los_POS	Instrument	micrOTOF-Q
Comment			

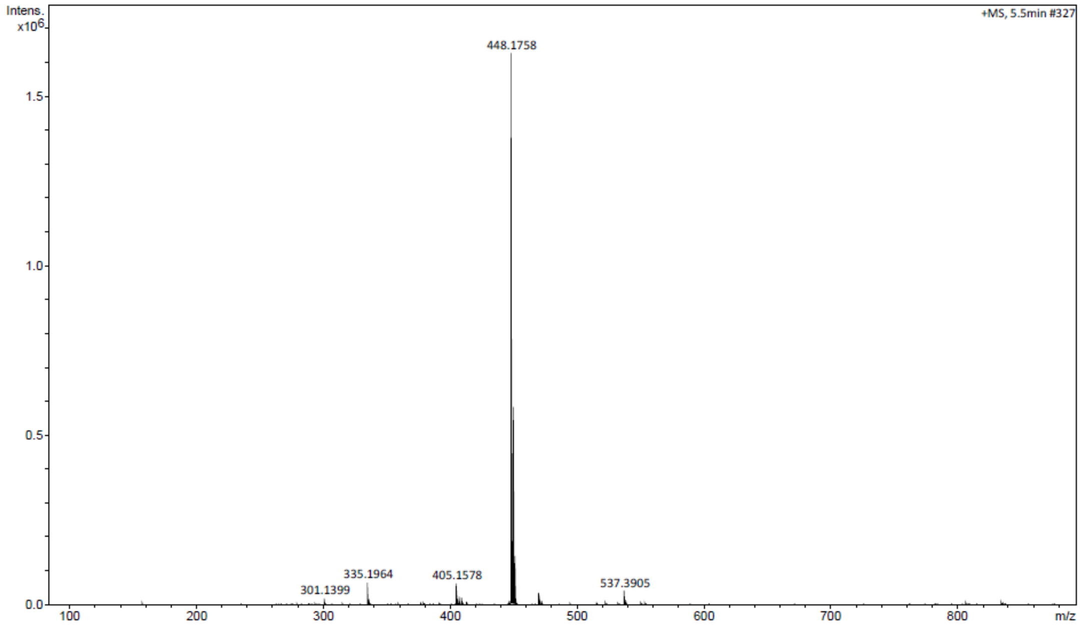

Bruker Compass DataAnalysis 4.3 printed: 12/5/2018 11:06:03 AM by: TOMAZ Page 1 of 1

Figure S5.

^{1}H NMR (500 MHz, CD_3CN) spectrum of 2-butyl-4-chloro-5-[((1*H*-1,2,3-triazol-4-yl)-(*N*,*N*-dimethyl-ammoniomethyl-trifluoroborate)methyl)methyl]-1-[(2′-(1*H*-tetrazol-5-yl) biphenyl-4-yl)methyl]-1*H*-imidazole (**AMBF3Los**).

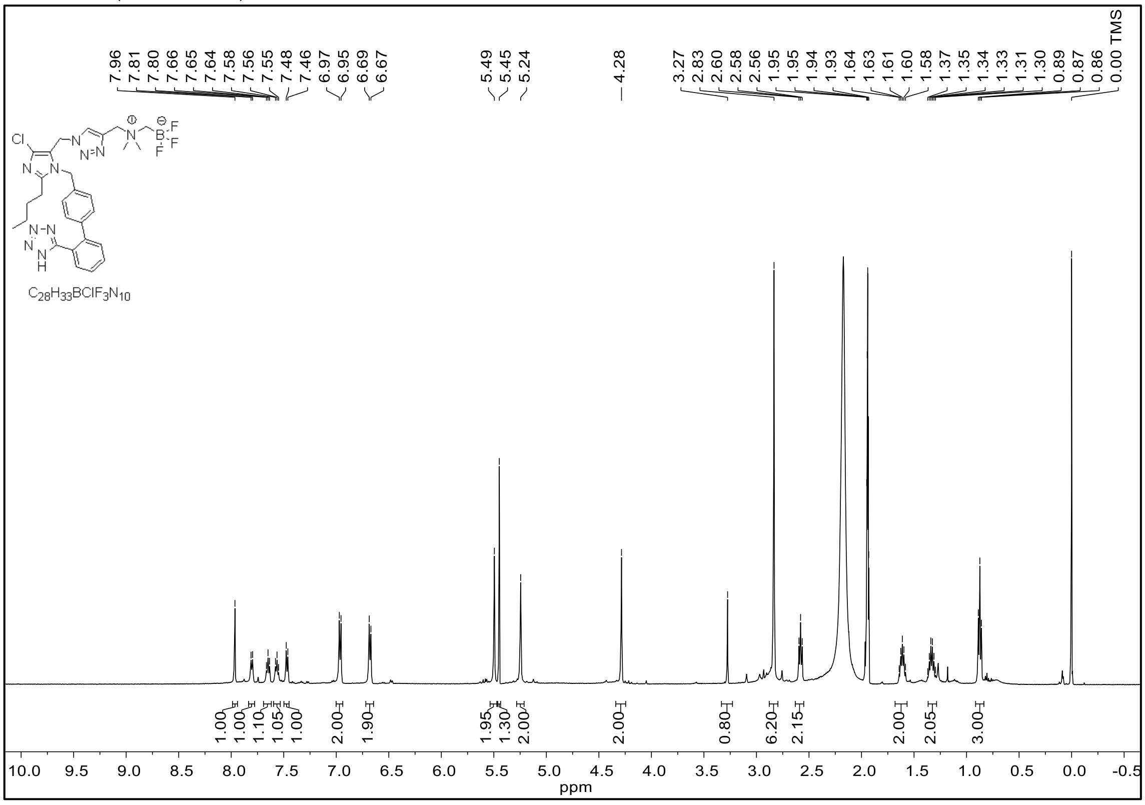

^{19}F NMR (470 MHz, CD_3CN) spectrum of **AMBF3Los**

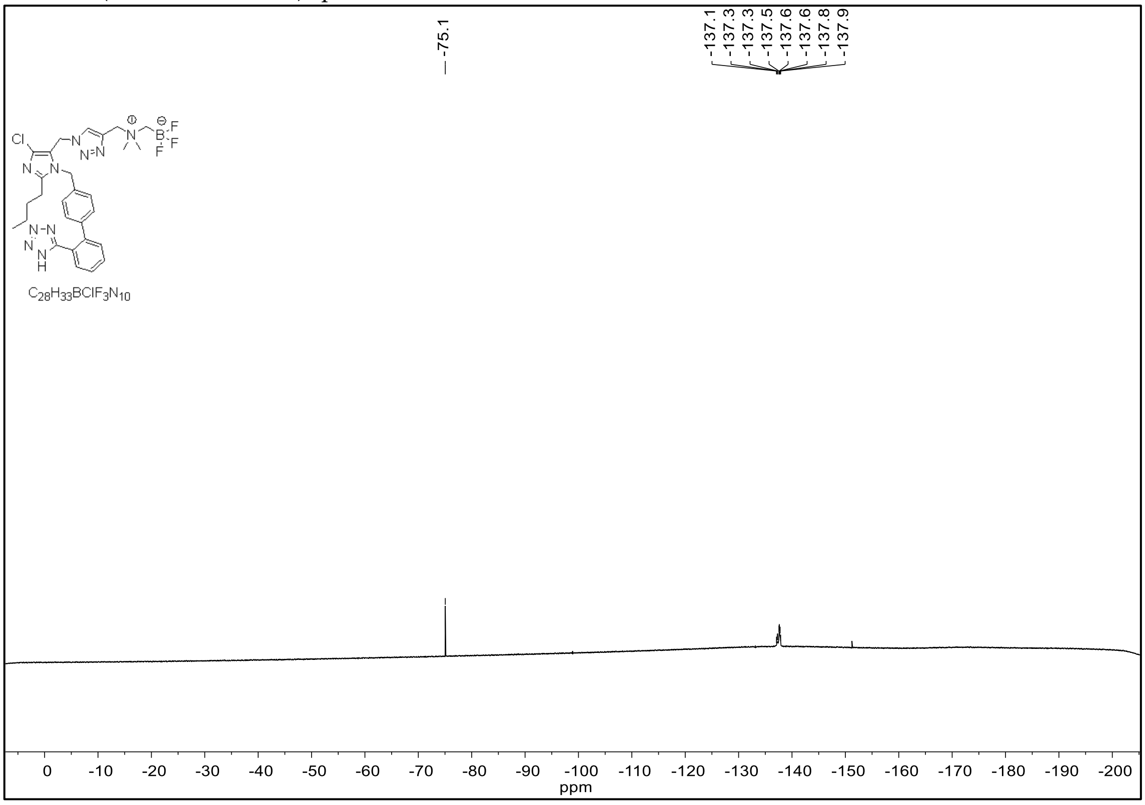

^{13}C NMR (75 MHz, CD_3CN) spectrum of **AMBF3Los**.

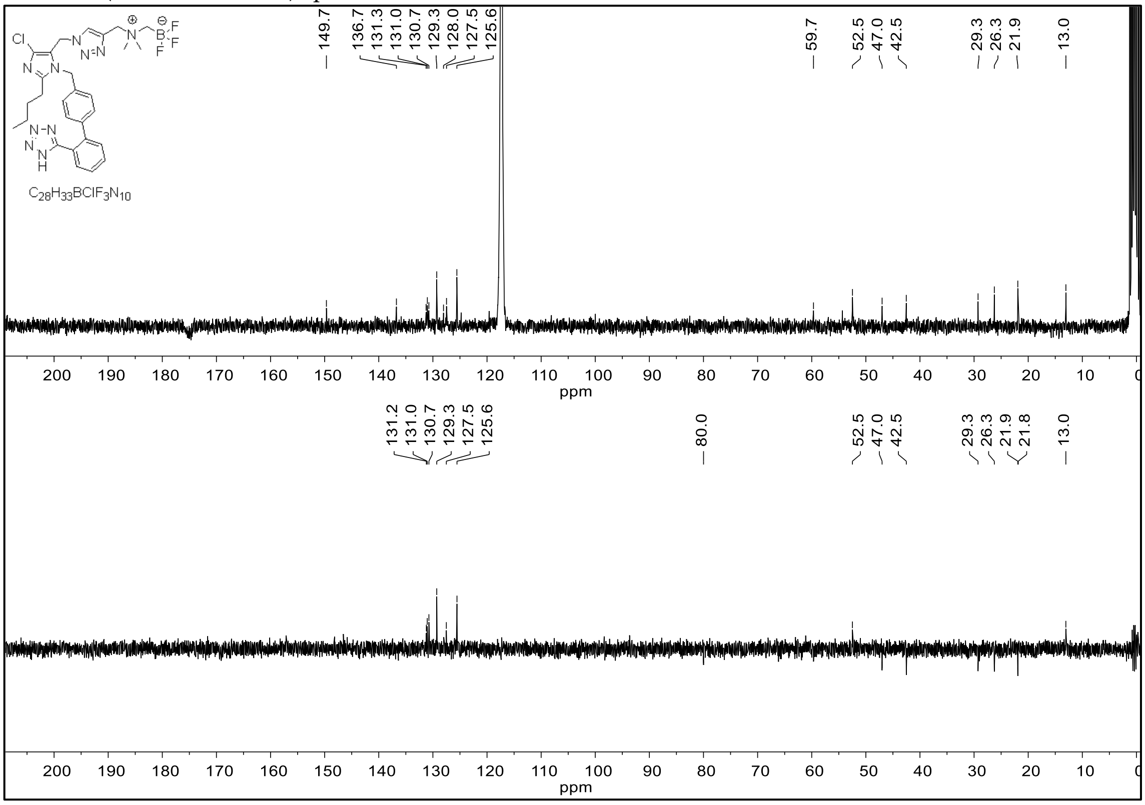

HMQC (300 MHz, CD_3CN) spectrum of **AMBF$_3$Los**.

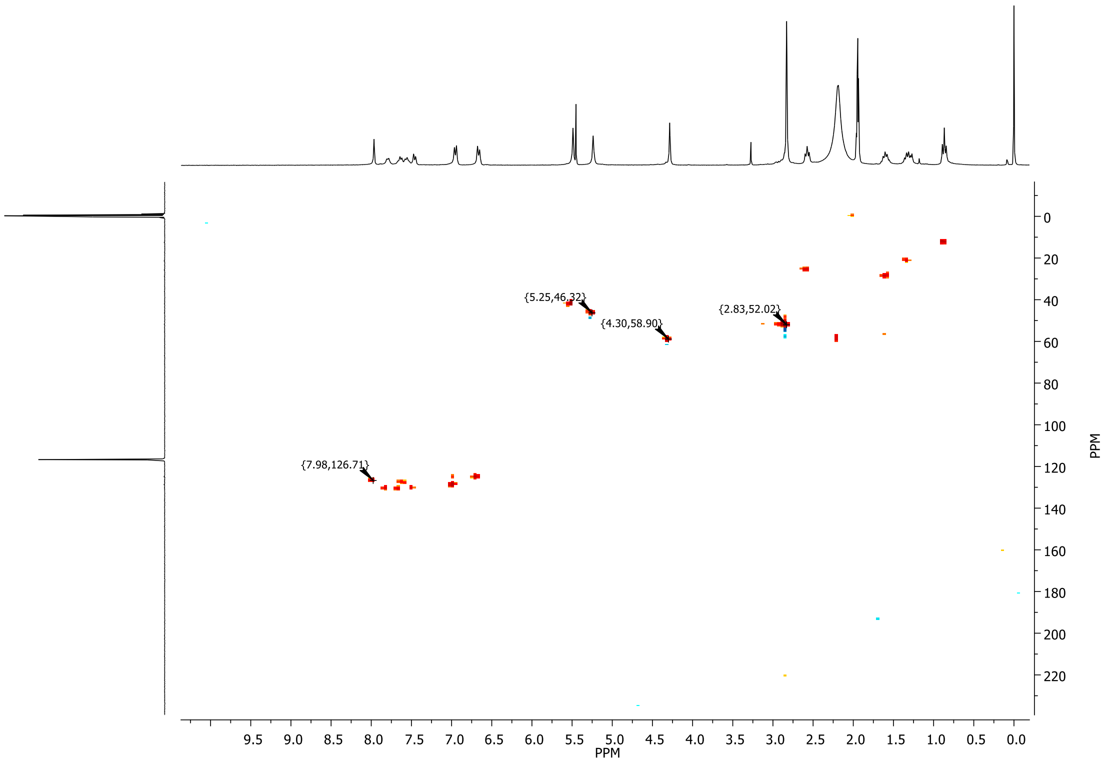

HMBC (300 MHz, CD_3CN) spectrum of **AMBF$_3$Los**.

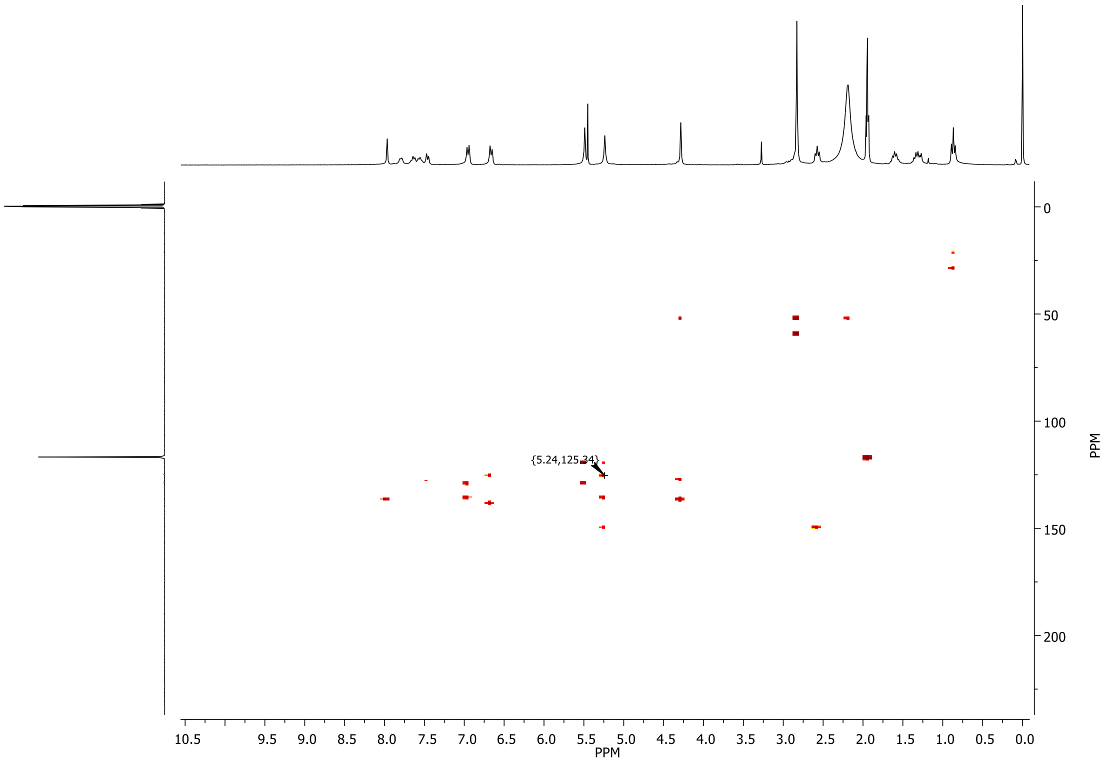

HRMS (ES^+): *m*/*z* $[M+H]^+$ calculated for $C_{28}H_{34}BClF_3N_{10}$: 613.2702; found: 613.2693 (**AMBF$_3$Los**).

Generic Display Report

Analysis Info

Analysis Name	D:\Data\PROF. IVONE\BF 3_Los_POSITIVO.d	Acquisition Date	12/3/2018 8:48:54 AM
Method	Tune_Low_Tomaz_Pos_1300u_Willian 1.m	Operator	TOMAZ
Sample Name	BF 3_Los_POSITIVO	Instrument	micrOTOF-Q
Comment			

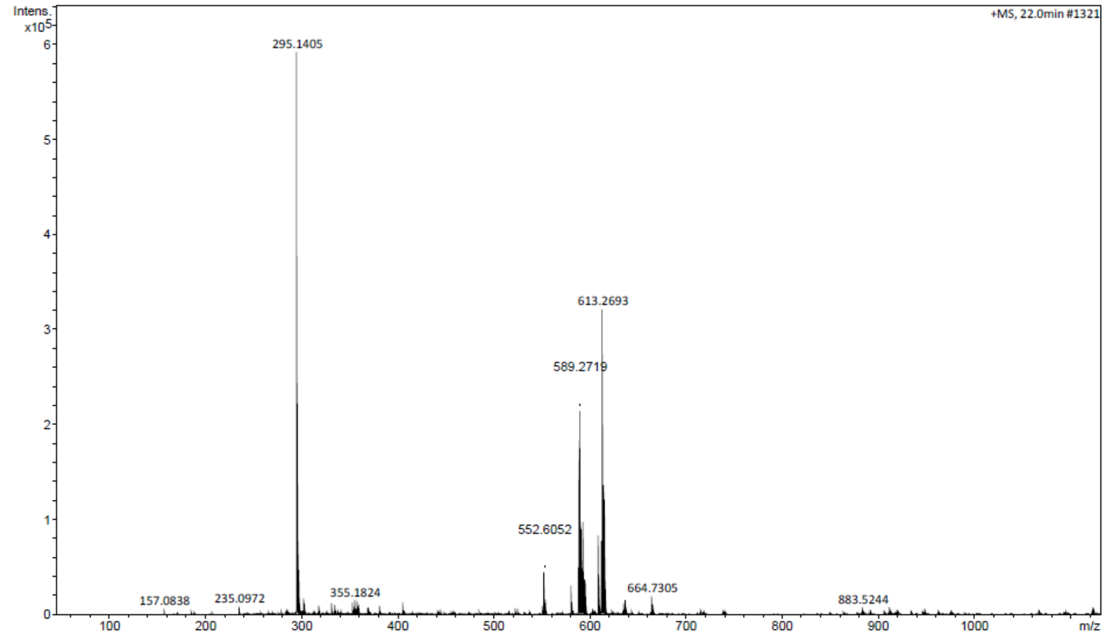

Bruker Compass DataAnalysis 4.3 printed: 12/5/2018 11:02:44 AM by: TOMAZ Page 1 of 1

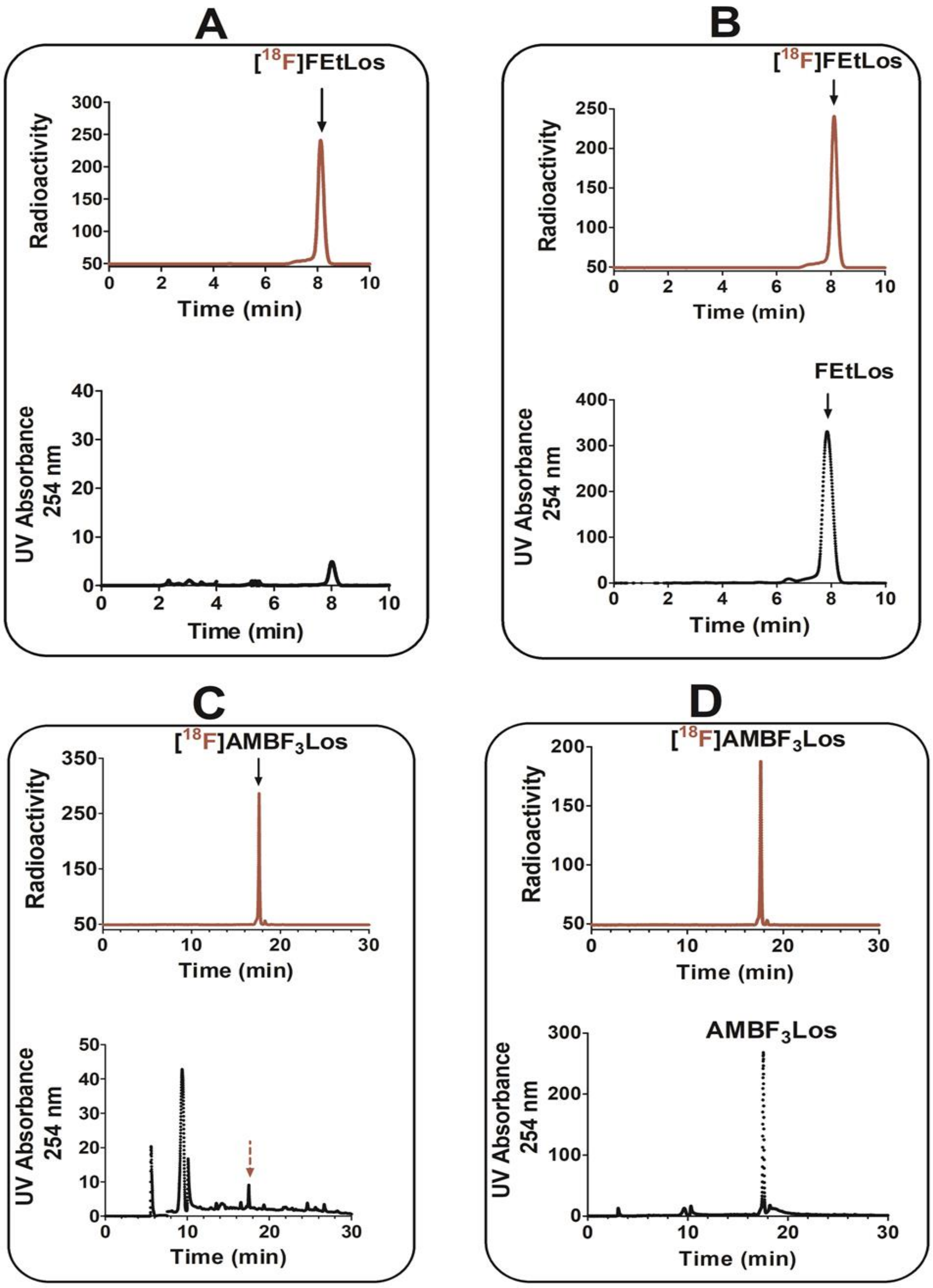

Figure S6. Quality control of the ^{18}F-labeled compounds. **A**. Representative chromatogram of the final formulation of **[^{18}F]FEtLos**. **B**. Representative chromatogram of **[^{18}F]FEtLos** after co-injection with the cold compound **FEtLos**. **C**. Representative chromatogram of the final formulation of **[^{18}F]AMBF$_3$Los**. **B**. Representative chromatogram of **[^{18}F]AMBF$_3$Los** after co-injection with the cold compound **AMBF$_3$Los**. Analytical HPLC conditions of 1b: solvent A: 0.1 % TFA water, solvent B: MeCN, 55:45 A/B, 1 mL/min. Analytical HPLC conditions of 8b: solvent A: 0.1 % TFA water, solvent B: MeCN, 0-100% B, 1 mL/min.

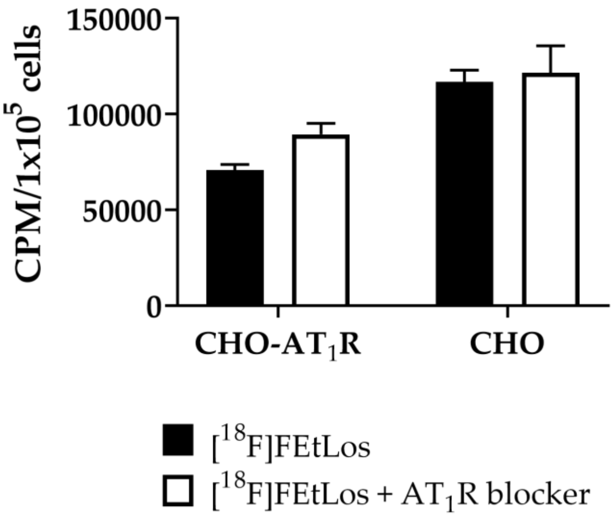

Figure S7. In vitro binding assay. Cells seeded in 6-well plates were incubated with **[^18F]FEtLos** for 60 minutes at 4 °C in presence (white bars, n=3) or absence (black bars, n=3) of the AT_1R blocker losartan (100 µM / well). Graph shows the mean ± SD of three independent experiments (n=3). The blocking effect was analyzed using the one unpaired t-test (multiple t tests); non-significant blocking effect was obtained.

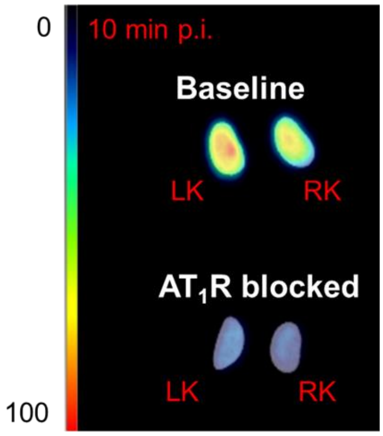

Figure S8. Ex vivo **[^18F]FEtLos** binding assay. Representative image of the *ex vivo* µPET/CT imaging of Balb/c Nude mice kidneys 10 minutes after **[^18F]FEtLos** intravenous injection in the absence (baseline) or presence of losartan potassium (AT_1R blocked). LK: left kidney; RK: right kidney.

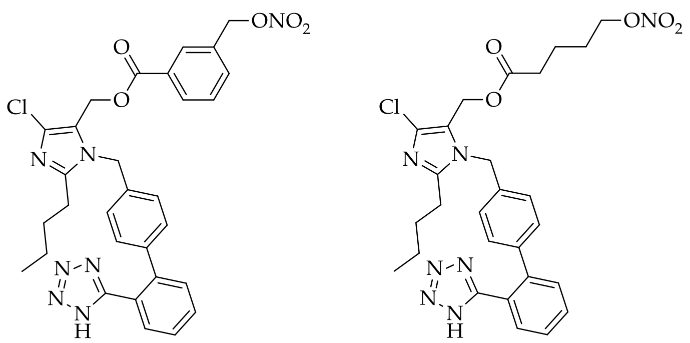

Figure S9. Structure of NO-releasing derivatives of losartan as cardiovascular drugs with vasorelaxing affects and AT_1R-antagonist activity [1]

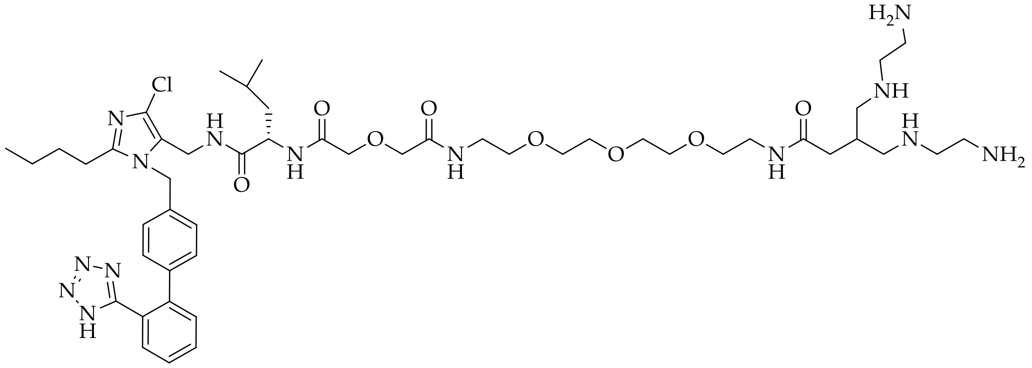

Figure S10. Structure of chelate-coupled losartan-Leucine-Diglycoloyl-Tetraethyleneglycol-Tetraamine for radiolabeling with ^{99m}Tc and molecular SPECT imaging in cardiology [2]

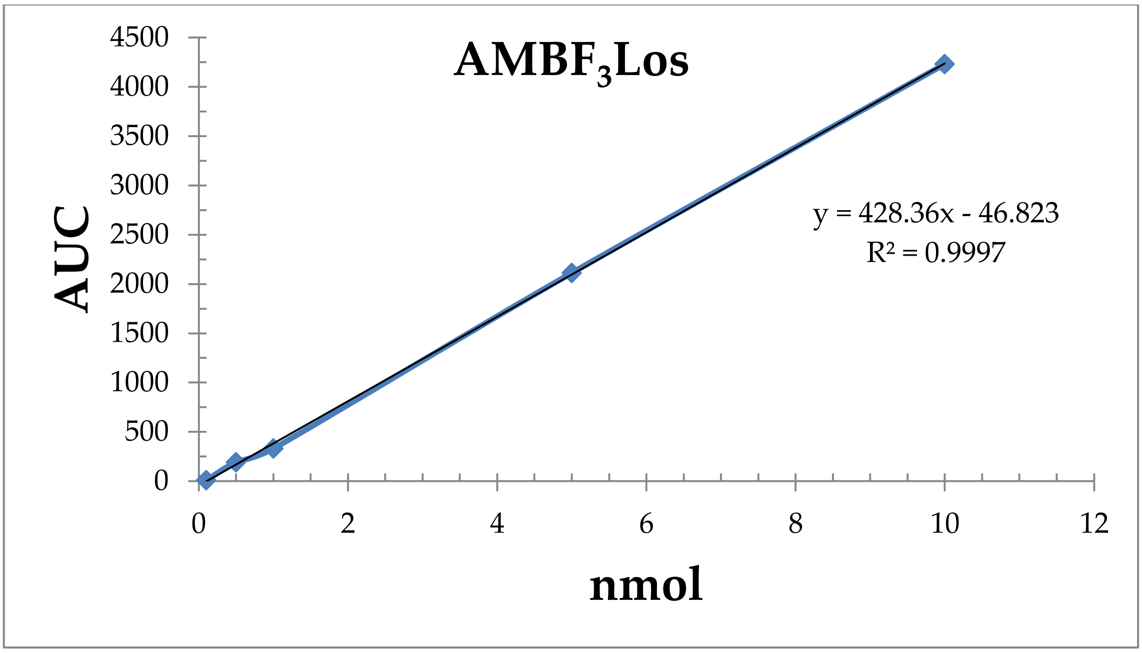

Figure S11. Calibration curve for determining the molar activity of [^{18}F]$AMBF_3Los$. The curve represent the correlation between the area under the curve (AUC), recorded from the analytical HPLC profile at 254 nm, and the amount (nmol) of the product injected onto analytical HPLC.

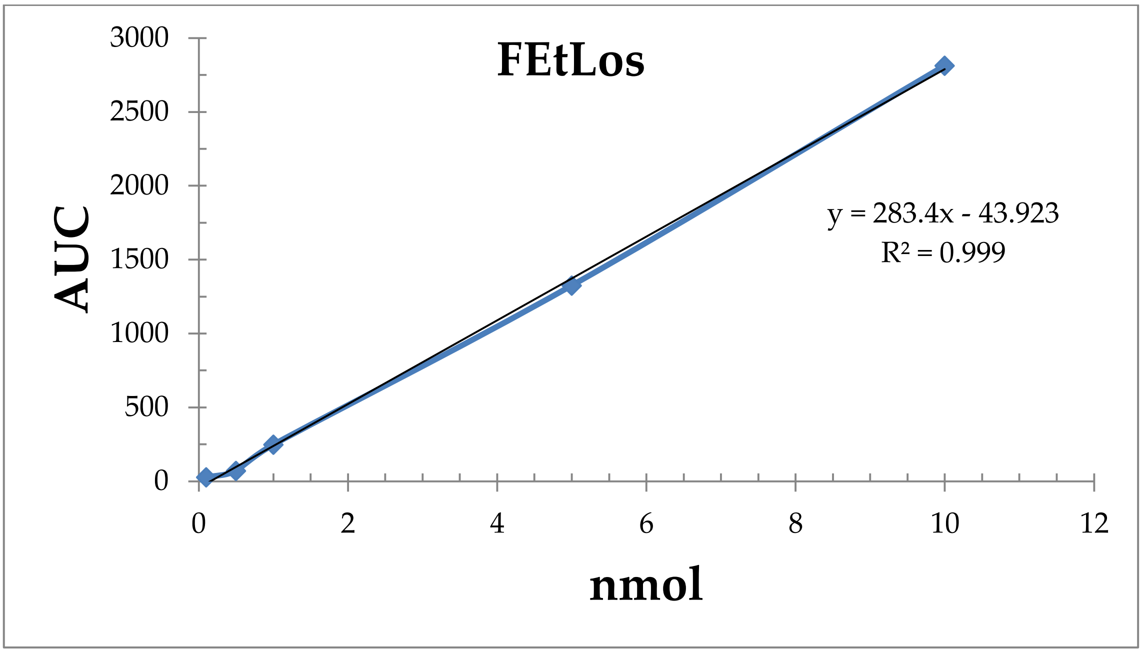

Figure S12. Calibration curve for determining the molar activity of [^{18}F]FEtLos. The curve represent the correlation between the area under the curve (AUC), recorded from the analytical HPLC profile at 254 nm, and the amount (nmol) of the product injected onto analytical HPLC.

Supplemental material and methods

1. Binding assays in cells with **[^{18}F]FEtLos**

CHO-AT1R and CHO cells (2 x 10^5) were plated in a 6-well plate overnight and then incubated for one hour at 4ºC with 10 µCi (370 kBq) of **[^{18}F]FEtLos** per well, in presence or absence of the AT_1R blocker (losartan potassium (100 µM / well) in PBS). At the end of the incubation period, the supernatant was aspirated and cells were washed six times with ice-cold PBS, and further removed with cell scraper and transferred to a gamma counter tube. The activity in the cells was counted in a Cobra II gamma counter (Packard).

2. *Ex vivo* **[^{18}F]FEtLos** binding assay

2.1. Animals

All the experiments were approved (Nº 152/15/CEUA -IPEN/SP) by the institutional animal care committee at IPEN (São Paulo, Brazil). Female immunodeficient Balb/c Nude mice were obtained from an in-house breeding colony in the Animal Resource Centre at IPEN. Mice were always divided into baseline and AT_1R blocked groups to assess the AT_1R binding specificity of **[^{18}F]FEtLos** with the co-injection of the AT_1R blocker losartan (70 mg/kg [3], dissolved in PBS).

2.2. *Ex vivo* µPET/CT imaging

The AT_1R binding specificity of **[^{18}F]FEtLos** was evaluated by *ex vivo* µPET/CT imaging of healthy Balb/c Nude mice kidneys after ten minutes of intravenous injection of [^{18}F]FEtLos with a dose of 8 ± 3 MBq (218 ± 70 µCi), in the absence (baseline) or presence of losartan potassium (AT_1R blocked). Mice were euthanized at ten minutes post injection by cervical dislocation, and the kidneys were harvested, rinsed in PBS and imaged with an Albira µPET/SPECT/CT imaging system (Brucker Corporation, Spain). The *ex vivo* µPET/CT imaging was recorded using a FOV (field of view) of 60 mm and 15 min for PET scan, and 35 kV and 400 µA for CT scan.

References

1. Breschi, M.C.; Calderone, V.; Digiacomo, M.; Martelli, A.; Martinotti, E.; Minutolo, F.; Rapposelli, S.; Balsamo, A. NO-sartans: a new class of pharmacodynamic hybrids as cardiovascular drugs. J Med Chem 2004, 47, 5597-5600.
2. Verjans, J.W.; Lovhaug, D.; Narula, N.; Petrov, A.D.; Indrevoll, B.; Bjurgert, E.; Krasieva, T.B.; Petersen, L.B.; Kindberg, G.M.; Solbakken, M., et al. Noninvasive imaging of angiotensin receptors after myocardial infarction. JACC Cardiovasc Imaging 2008, 1, 354-362.
3. Coulson, R.; Liew, S.H.; Connelly, A.A.; Yee, N.S.; Deb, S.; Kumar, B.; Vargas, A.C.; O'Toole, S.A.; Parslow, A.C.; Poh, A., et al. The angiotensin receptor blocker, Losartan, inhibits mammary tumor development and progression to invasive carcinoma. Oncotarget 2017, 8, 18640-18656.
